# Transcriptional signatures of participant-derived neural progenitor cells and neurons implicate altered Wnt signaling in Phelan-McDermid syndrome and autism

**DOI:** 10.1186/s13229-020-00355-0

**Published:** 2020-06-19

**Authors:** Michael S. Breen, Andrew Browne, Gabriel E. Hoffman, Sofia Stathopoulos, Kristen Brennand, Joseph D. Buxbaum, Elodie Drapeau

**Affiliations:** 1 Seaver Autism Center for Research and Treatment, Icahn School of Medicine at Mount Sinai, New York, USA; 2 Department of Psychiatry, Icahn School of Medicine at Mount Sinai, New York, USA; 3 Department of Genetics and Genomic Sciences, Icahn School of Medicine at Mount Sinai, New York, USA; 4 Nash Family Department of Neuroscience, Icahn School of Medicine at Mount Sinai, New York, USA; 5 Icahn Institute for Data Science and Genomic Technology, Icahn School of Medicine at Mount Sinai, New York, USA; 6 Friedman Brain Institute, Icahn School of Medicine at Mount Sinai, New York, USA; 7 Mindich Child Health and Development Institute, Icahn School of Medicine at Mount Sinai, New York, USA; 8 Pamela Sklar Division of Psychiatric Genomics, Icahn School of Medicine at Mount Sinai, New York, NY 10029 USA

**Keywords:** Neural progenitor cells, Neurons, Stem cells, RNA-sequencing, Autism spectrum disorder

## Abstract

**Background:**

Phelan-McDermid syndrome (PMS) is a rare genetic disorder with high risk of autism spectrum disorder (ASD), intellectual disability, and language delay, and is caused by 22q13.3 deletions or mutations in the *SHANK3* gene. To date, the molecular and pathway changes resulting from *SHANK3* haploinsufficiency in PMS remain poorly understood. Uncovering these mechanisms is critical for understanding pathobiology of PMS and, ultimately, for the development of new therapeutic interventions.

**Methods:**

We developed human-induced pluripotent stem cell (hiPSC)-based models of PMS by reprogramming peripheral blood samples from individuals with PMS (*n* = 7) and their unaffected siblings (*n* = 6). For each participant, up to three hiPSC clones were generated and differentiated into induced neural progenitor cells (hiPSC-NPCs; *n* = 39) and induced forebrain neurons (hiPSC-neurons; *n* = 41). Genome-wide RNA-sequencing was applied to explore transcriptional differences between PMS probands and unaffected siblings.

**Results:**

Transcriptome analyses identified 391 differentially expressed genes (DEGs) in hiPSC-NPCs and 82 DEGs in hiPSC-neurons, when comparing cells from PMS probands and unaffected siblings (FDR < 5%). Genes under-expressed in PMS were implicated in Wnt signaling, embryonic development, and protein translation, while over-expressed genes were enriched for pre- and postsynaptic density genes, regulation of synaptic plasticity, and G-protein-gated potassium channel activity. Gene co-expression network analysis identified two modules in hiPSC-neurons that were over-expressed in PMS, implicating postsynaptic signaling and GDP binding, and both modules harbored a significant enrichment of genetic risk loci for developmental delay and intellectual disability. Finally, PMS-associated genes were integrated with other ASD hiPSC transcriptome findings and several points of convergence were identified, indicating altered Wnt signaling and extracellular matrix.

**Limitations:**

Given the rarity of the condition, we could not carry out experimental validation in independent biological samples. In addition, functional and morphological phenotypes caused by loss of *SHANK3* were not characterized here.

**Conclusions:**

This is the largest human neural sample analyzed in PMS. Genome-wide RNA-sequencing in hiPSC-derived neural cells from individuals with PMS revealed both shared and distinct transcriptional signatures across hiPSC-NPCs and hiPSC-neurons, including many genes implicated in risk for ASD, as well as specific neurobiological pathways, including the Wnt pathway.

## Introduction

Phelan-McDermid syndrome (PMS) is one of the most penetrant and more common single-locus causes of autism spectrum disorder (ASD), accounting for ca. 1% of ASD diagnoses [1–3]. PMS is caused by heterozygous 22q13.3 deletions or mutations leading to haploinsufficiency of the *SHANK3* gene [2, 4–6]. Clinical manifestations of PMS include ASD, global developmental delay, severe to profound intellectual disability (ID), motor abnormalities, delayed or absent speech, and epilepsy [2, 6, 7]. *SHANK3* is a scaffolding protein of the postsynaptic density of excitatory synapses and plays a critical role in synaptic function, being a key component in the integration of glutamatergic synaptic signaling [8–14]. A fundamental knowledge gap separates the well-defined clinical impact of *SHANK3* mutations and the early developmental, molecular and cellular mechanisms leading to these neurodevelopmental phenotypes.

Several studies have utilized murine models to explore the molecular consequences of *SHANK3*-deficiency. We and others have shown that mice with a disruption in *Shank3* have altered glutamatergic signaling and synaptic dysfunction, as well as altered motor, social, and repetitive behaviors [11, 15–26]. Studies on a genetically modified Shank3 rat model showed deficits in attention and in long-term social memory, which were attributable to reduced synaptic plasticity in the hippocampal-medial prefrontal cortex pathway [27, 28]. Many of the features of these rodent models reflect deficits similar to those observed in PMS. However, although animals and humans share homologous genes, pathways, and networks, rodents may have limits as models of human neurodevelopment. Specifically, the biological context and integration of molecular pathways differ across species, which can pose an obstacle for drug development and discovery.

The generation of neuronal cultures from human-induced pluripotent stem cells (hiPSCs) has the potential to create translatable and experimentally tractable human neuronal models [29, 30]. hiPSCs can be derived directly from participant cells and reprogrammed to differentiate into target cell types of interest, recapitulating the early stages of neurodevelopment in vitro, all while retaining the genetics of the original donor. In several studies, hiPSC-derived neurons have been examined from individuals with PMS and show a reduction in the number of synapses in *SHANK3*-deficient neurons, together with smaller cell bodies, more extensively branched neurites, reduced motility, impaired dendritic arborization, and major deficits in excitatory, but not inhibitory, synaptic activity [31–36]. Isogenic comparisons of CRISPR-engineered heterozygous and homozygous *SHANK3* mutations demonstrated that *SHANK3*-deficiency causes functionally impaired hyperpolarization-activated cation currents, likely through its ability to interact with and organize the hyperpolarization-activated cyclic nucleotide-gated channels that mediate *I*_h_ currents [37]. Some studies indicate that excitatory synaptic transmission in PMS neurons can be corrected by restoring *SHANK3* expression, by treating neurons with IGF-1, or by pharmacologically and genetically activating Akt or inhibiting the Cdc2-like kinase 2 activity [31, 34, 35]. Amelioration of deficits associated with *SHANK3* haploinsufficiency have also been demonstrated by treating hiPSCs with lithium or valproic acid [34].

Overall, both the animal and hiPSC-based studies consistently confirm that, at the neurophysiological level, PMS leads to a disruption in glutamatergic signaling. A next practical step would be to identify consistent molecular changes in PMS, specifically, the repertoire of genes and molecular pathways that are altered in expression as a consequence of *SHANK3*-deficiency. We and others have successfully used hiPSCs as reliable models to study transcriptional and synaptic changes in syndromic and idiopathic forms of a variety of neurodevelopmental disorders [38–40], with the ultimate goal of guiding novel therapeutic approaches. For example, transcriptome-wide screening in hiPSCs and maturing hiPSC-derived neurons (hiPSC-neurons) from individuals with ASD and macrocephaly demonstrates ASD-specific temporal dysregulation, which appears to underlie the observed in vitro cellular phenotypes [40]. Similarly, CRISPR knockout of several top ranked ASD risk genes in hiPSCs and hiPSC-neurons reveal points of convergence for altered gene expression patterns [41], which underlie reduced synaptic activity in these cell types. Collectively, these transcriptional changes underscore the importance of studying hiPSC-derived neural cells to capture critical changes in early developmental molecular pathways. In the current study, we hypothesized that a better understanding of the transcriptional mechanisms of *SHANK3*-defiency in PMS participant derived hiPSC-derived neural progenitor cells (hiPSC-NPCs) and hiPSC-neurons would complement prior studies on functional and morphological phenotypes, and inform the search for approaches to ameliorate the previously reported neurobiological and neurophysiological deficits due to haploinsufficiency of *SHANK3.*

The overarching objective of the current study was to identify the transcriptional signatures of *SHANK3*-deficiency in hiPSC-NPCs and hiPSC-neurons by comparing gene expression between PMS probands (*n* = 7) and unaffected siblings (*n* = 6), using genome-wide RNA-sequencing (RNA-seq). A multi-step analytic approach was applied to (1) confirm the developmental specificity of our hiPSC neural cells; (2) quantify the variance in hiPSC-NPC and hiPSC-neuron transcriptome data that is explained by differences in neural cell types, individual donors, and other relevant factors; and (3) identify and characterize candidate genes, molecular pathways, and co-regulatory networks associated with PMS in hiPSC-NPCs and hiPSC-neurons. We identify molecular pathways that both inform pathobiological mechanisms in PMS and suggest approaches for interventions.

## Materials and methods

### Participants

The study includes 13 participants (Table 1, seven probands and six unaffected siblings) enrolled at the Seaver Autism Center for Research and Treatment at the Icahn School of Medicine at Mount Sinai. Individuals were referred through the Phelan-McDermid Syndrome Foundation, ongoing research studies, and communication between families. The study was approved by the Program for the Protection of Human Subjects at the Icahn School of Medicine at Mount Sinai. Parents or legal guardians provided informed consent for participation and publication.

**Table 1:** Genetic and demographic information for PMS probands and unaffected siblings

	PMS proband		Unaffected sibling		Genetic and genomic characterization of SHANK3 mutation			
Family ID	Age (yrs)	Sex	Age (yrs)	Sex	Mutation type	Minimal deletion size	Chromosomal location	Genes within deletion on chr22
1	5	F	3	F	Frameshift	N/A	chr22:51160837-51160839 GCC/G	*SHANK3*
2	13	F	6	F	Deletion	42 kb	chr22:51,132,839-51,175,792	*SHANK3*
3	25	F	19	M	Deletion	43 kb	chr22:51,132,839-51,176,002	*SHANK3*
4	3	M	1	F	Deletion	62 kb	chr22:51,121,360-51,183,840	*SHANK3, ACR*
5	3	M	6	F	Deletion	85 kb	chr22:51,086,931-51,172,228	*SHANK3, ACR, RABL2B*
6	4	F	6	M	Deletion	4.98 Mb	chr22:46316673-51,304,566	109 genes (see Table S1)
7	9	F	12	M	Deletion	6.9 Mb	chr22:44321641-51,304,566*	167 genes (see Table S1)

hiPSC-NPCs could not be generated for unaffected sibling from family ID 2. Family 6 is of Asian ancestry and the remaining families are of European ancestry

### Genetic findings

The mutation in patient 1 was identified through clinical WES by the Medical Genetics Laboratory at the Baylor College of Medicine. Deletions in patients 2–7 were identified as follows: patient 2, FISH and chromosome microarray (CMA) by Signature Genomics; patient 3, CMA by the Genetics Laboratory at the University of Oklahoma Health Sciences Center; patient 4, FISH by Quest Diagnostic and CMA by the Shaare Zedek Medical Center, Jerusalem; patient 5, CMA at the UCSF Benioff Children’s Hospital Oakland; patient 6, CMA by the Mount Sinai Genetic Testing Laboratory; and patient 7, karyotyping and custom OGT 22q array by cytogenic laboratory of the Greenwood Genetic Center.

Variants were annotated according to the Human Genome Variation Society guidelines. As reported previously, the human genome reference assembly (GRCh37/hg19 and GRCh38/hg38) is missing the beginning of exon 11 (NM_033517.1:c.1305_1346, 5′-cccgagcgggcccggcggccccggccccgcgcccggccccgg-3′, coding for 436-PSGPGGPGPAPGPG-449). We numbered nucleotide and amino acid positions according to the *SHANK3* RefSeq mRNA (NM_033517.1) and protein (NP_277052.1) sequence, in which this mistake has been corrected. Variants were interpreted according to the American College of Medical Genetics and Genomics (ACMG) guidelines.

### hiPSC generation

Blood samples were collected from all participants and used for both DNA isolation (DNeasy Blood and Tissue Kit, QIAGEN) and peripheral blood mononuclear cells (PBMCs) extraction (BD Vacutainer CPT Mononuclear Cell Preparation Tubes with Sodium Heparin, BD Biosciences) according to manufacturer’s instructions. PBMCs were cultured for 9 to 12 days in an erythroblast enrichment medium [42] to expand the erythroblast population and 2.5 × 10^5^ cells were transduced using recombinant Sendai viral vectors (Cytotune-iPSC 2.0^TM^, Thermofisher scientific), expressing the four reprogramming factors *Oct4*, *Sox2*, *Kfl4*, and *c*-*Myc*, according to manufacturer’s instructions. After 3 days, transduced cells were plated on irradiated mouse embryonic fibroblast (MEFs; Fisher A34961) and grown for 2 to 3 weeks in hESC medium until the emergence of individual colonies. Live hiPSCs were labeled by Tra-1-60 immunostaining (R&D systems) and positive clones were manually picked and grown on MEFs using hiPSC hiPSC medium (DMEM/F12, 20% knockout serum, 1X MEM-NEAA, 1X L-glutamine, 1X pen/strep, 0.1 mM 2-mercaptoethanol, 20 ng/mL b-FGF). After reaching passage 10, hiPSC colonies were transitioned to feeder-free conditions using Matrigel-coated plates (Corning) and mTeSR1 medium (Stem Cell Technology) and up to three clones per individual were validated, expanded and cryopreserved.

### hiPSC validation

All hiPSC lines were assessed for chromosomal abnormalities by performing karyotyping (WiCell). Their identity was confirmed using short-tandem repeat (STR) analysis (WiCell) and comparison with the donor’s blood DNA. The potential for self-renewal and pluripotency of the hiPSC lines was assessed by utilizing hiPSC RNA with the Taqman hPSC Scorecard Assay (Thermo Fisher, A15870). For pluripotency, RNA was isolated after random differentiation of hiPSCs into embryoid bodies [43] to generate the three primary germ layers and assessed utilizing EB RNA with the Taqman hPSC Scorecard Assay. Full elimination of the Sendai virus vectors was confirmed by immunostaining and with the Taqman hPSC Scorecard Assay. The e-Myco Mycoplasma PCR Detection Kit (Bulldog Bio, 25233) and the MycoAlert Mycoplasma Detection Kit (Lonza, LT07-118) were used to ensure that all the cells used in this study were mycoplasma-free.

### Generation of neuronal progenitor cells

Neural progenitor cells (hiPSC-NPCs) were induced from passage 16 to 18 hiPSCs using the PSC neural induction medium (Invitrogen) according to the manufacturer’s protocol. They were maintained in PSC neural expansion medium up to passage 4 and then transferred to NPC medium (DMEM/F12, N2, B27 without retinoic acid, 1 μg/mL natural mouse laminin, 20 ng/mL FGF2). At passage 5, NPCs were labeled with SOX2 (Santa Cruz SC-17320, 1:100) and Nestin (ThermoFisher MA1-110, 1:200) antibodies to confirm their cellular identity and validated NPCs were cryopreserved. For RNA isolation, NPCs at passage 6 were seeded into 12-well plates at a density of 750,000 cells per well and harvested on the seventh day after plating using RNA-Bee (BioConnect, CS-104B) and RNA was extracted according to the manufacturer’s protocol.

### Generation of forebrain neurons

For neuronal differentiation, NPCs at passage 6 were plated on to Matrigel-coated 6-well plates at a density of 200,000 cells per well in neural differentiation medium (DMEM/F12, N2, B27 without retinoic acid, 1 μg/mL natural mouse laminin, 500 μg/mL Dibutyryl cyclic-AMP, 20 ng/mL BDNF, 20 ng/mL GDNF, 200 nM l-Ascorbic Acid [44]). Neurons were cultured for 4, 6, or 8 weeks with replacement of two-thirds of the medium every 3 days. For immunostaining, additional neurons were plated on Matrigel-coated coverslips and similarly processed. At each time-point, immunostaining with MAP2 (Millipore MAB3418, 1:500) and Beta-3-Tubulin (Abcam ab18207, 1:1000) was performed for all samples to confirm their neuronal identity and cells for RNA sequencing were harvested using RNA-Bee. RNA was extracted using the same procedures as described for NPC samples. Here, the term “hiPSC-neurons” refers to mixed forebrain neuron cultures.

### RNA isolation, library preparation, and sequencing

RNA samples were processed for RNA-sequencing to form two groups: (1) a larger discovery set; and (2) a smaller replication set. For the discovery set, 39 hiPSC-NPC and 41 hiPSC-neuron RNA samples underwent RNA-sequencing. For the replication set, 21 hiPSC-neuron RNA samples collected at 6 weeks underwent RNA-sequencing. The integrity for each RNA sample was measured using the Agilent 2100 Bioanalyzer (Agilent, Santa Clara, CA, USA). All RNA integrity numbers (RINs) were greater than 8 (RIN: 9.59 ± 0.43). RNA samples were purified using PolyA selection, and the Illumina TruSeq Stranded Total RNA kit (Ilumina, San Diego, CA, USA) was used for library preparation, according to the manufacturer instructions. All indexed RNA libraries were pooled and sequenced using long read paired-end chemistry (2 × 150 bp) at an average read depth of ~ 50 M reads per sample using the Illumina HiSeq2500. Resulting short reads with Illumina adapters were trimmed and low-quality reads were filtered using TrimGalore (*--illumina* option) [45]. All high-quality reads were then processed for alignment using the hg38 reference and the ultrafast universal RNA-seq aligner STAR (v2.5.1) [46] with default parameters. Mapped bam files were sorted using Samtools and short read data were quantified using featureCounts [47] with the following parameters: -T 5, -t exon, and -g gene_id. Subsequently, all read counts were exported and all downstream analyses were performed in the R statistical computing environment.

### RNA-seq data pre-processing and quality control

Raw count data was subjected to non-specific filtering to remove low-expressed genes that did not meet the requirement of a minimum of two counts per million (cpm) in at least ~ 40% of samples. This filtering threshold was applied to hiPSC-NPCs and hiPSC-neurons separately. All expression values were converted to log_2_ RPKM and subjected to unsupervised principal component analysis (PCA) to identify and remove outlier samples that lay outside 95% confidence intervals from the grand averages as well as samples with aberrant X-inactivation gene expression profiles. The following high-quality RNA-seq samples passed into all downstream analyses: from the discovery set, a total of 32 hiPSC-NPCs composed of seven PMS cases and six siblings, and a total of 41 hiPSC-neurons, composed of five PMS cases and five siblings; from the replication set, and a total of 17 hiPSC-neurons composed of five PMS cases and five siblings.

### Developmental specificity analysis

Two independent analyses were performed to confirm the developmental specificity of our hiPSC-NPC and hiPSC-neuronal gene expression data. First, we sought to confirm the developmental origin of our samples by integrating several RNA-seq data sets. from either postmortem brain tissue or hiPSC models, with our hiPSC gene expression data using a previously described approach [48]. A total of 15 independent studies were collected covering 2716 independent samples and 11,650 genes. All expression values were converted to log_2_ RPKM and collectively normalized using quantile normalization using the *limma* R package [49]. These data, along with our hiPSC expression data, were analyzed jointly and integrated using principal component analysis (PCA). Second, we sought to confirm that highly expressed genes in our current data set are indeed preferentially prenatally biased in expression, based on BrainSpan developmental RNA-seq data. We previously applied a linear regression model to 299 neocortical BrainSpan samples ranging from eight post-conceptual weeks to 40 years of age in order to characterize 22,141 genes as either prenatally or postnatally biased (log_2_FC > 0.1 and *q* < 0.05) or unbiased in expression (*q* > 0.05) [50]. The regression model generated a “prenatal effect” (*t-*statistic) of the log_2_ fold-change of prenatal versus postnatal transcript abundance. We leveraged these summary statistics to examine the top 1000 most expressed and top 1000 least expressed genes in both hiPSC-NPCs and hiPSC-neurons. Each gene set was examined to determine if the distribution of the fetal effect (i.e., t-statistics for each gene set) differed significantly from the entire neocortical background using a Wilcoxon signed rank test. The neocortical background was defined as genes which were simultaneously detected by RNA-seq in the current study as well as genes found to be expressed in the neocortex following quality control procedures.

### Cell type deconvolution analysis

The frequencies of neural cell types were estimated using Cibersort cell type deconvolution (https://cibersort.stanford.edu/) [51]. Cibersort relies on known cell subset specific marker genes to predict the proportions of cell types in heterogeneous bulk RNA-sequencing data. The method applies linear support vector regression, a machine learning approach that is robust compared to other methods with respect to noise, unknown mixture content, and closely related cell types. As input, we used a reference panel of single-cell RNA-sequencing data from the human fetal cortex [52]. Cell-specific gene signatures were curated using pre-defined cell clusters from the original publication covering four major cell types: (i) dividing intermediate progenitor cells (clusters 15–19); (ii) excitatory neurons (clusters 21–28); (iii) inhibitory neurons (clusters 38–46); and (iv) mixed glial cells (clusters 4, 6, 9, 19). Definitions for excitatory and inhibitory cell lineages in these data were defined in our previous work [50].

### Quantifying transcriptome variance explained by known factors

Following data quality control, outlier detection, and developmental specificity analysis (all described above), all gene expression values were normalized using VOOM normalization (a variance-stabilization transformation method) [49], and these data were used to carry out the remainder of downstream analyses. To understand the effects of various recorded factors on gene expression patterns, linear mixed effect models were applied to decompose the transcriptome variability into discrete percentages of variability attributable to multiple biological and technical sources of variation using the R package variancePartition [53]. For each gene, the percentage of gene expression variation attributable to differences in induced cell types (*i.e.*, hiPSC-NPCs versus hiPSC-neurons), individual as a repeated measure (i.e., inter-donor effects), family effects, PMS diagnosis, RIN, age, biological sex, sequencing batch, and variation in estimated cell type frequencies was computed. By properly attributing multiple sources of expression variation in this fashion, it is possible to identify and partially correct for some confounding variables in our differential gene expression analysis.

### eQTL enrichment analysis

We used our previously described approach [48] to examine the overlap between genes with eQTLs from the CommonMind Consortium and genes exceeding a variance percentage cutoff for a particular variable of interest in the current study. In brief, varianceParition analysis was applied to assign each gene a fraction of variance explained by a specific observed factor in the current analysis. A total of 40 different variance explained cutoff thresholds were examined and the overlap between genes with values exceeding this cutoff and the 2000 genes with the smallest *p* values from *cis*-eQTL analysis is evaluated. The overlap is computed for the observed data and 10,000 data sets with the variance percentages randomly permutated. At each cutoff where > 100 genes are represented, the fold enrichment is computed as the observed overlap over the permuted overlap.

### Differential gene expression analysis

Differential gene expression analyses were conducted using a moderated *t* test from the R package limma [49]. All analyses adjusted for the possible confounding influence of biological sex, sequencing batch, and RIN. Moreover, due to the repeated measures study design, where individuals are represented by multiple independent technical replicates, the duplicateCorrelation function was applied in the limma analysis and gene level significance values were adjusted for multiple testing using the Benjamini and Hochberg method to control the false discovery rate (FDR). Genes passing a FDR < 5% were labeled as showing significantly altered expression.

### Functional enrichment of differentially expressed genes

Functional annotation was assessed in two complementary ways. First, all differentially expressed genes (FDR < 5%) were functional annotated using the ToppFun module of ToppGene Suite software [54]. We explored Gene Ontology terms related to biological processes using a one-tailed hyper-geometric tested (Benjamini–Hochberg (BH) FDR corrected) to assess the significance of the overlap. Enrichment was examined separately for over-expressed and under-expressed genes. All terms must pass an FDR-corrected *p* value and a minimum of three genes per ontology were used as filters prior to pruning ontologies to less redundant terms. Second, we applied the camera function in the R package *limma* [49] to perform a competitive gene set test and to assess whether the genes in a given set are high or low ranked in terms of differential gene expression relative to genes that are not in the set. By nature of the test, genes expressed in the current data set are used as background set. The method leverages limma’s linear model framework, taking both the design matrix and contrast matrix and accommodates the observational-level weights from *voom* in the testing procedure. After adjusting the variance of the resulting gene set test statistic by a variance inflation factor that depends on the gene-wise correlation (which is set to 0.01 by default) and the size of the set, a *p* value is returned and adjusted for multiple testing.

### Protein-protein interaction networks

The STRING database v11.0 [55] was used to assess whether differentially expressed genes were enriched for direct protein–protein interactions (PPIs) and to identify key genes mediating the regulation of multiple targets. For these analyses, our signature query of PMS-associated genes (FDR< 5%) were used as input. STRING implements a scoring scheme to report the confidence level for each direct PPI (low confidence: < 0.4; medium: 0.4–0.7; high: > 0.7). We used a combined STRING score > 0.4. Hub genes within the PPI network are defined as those with the highest degree of network connections. We further used STRING to test whether the number of observed PPIs were significantly more than expected by chance using a nontrivial random background model. For visualization, the STRING network was imported into Cytoscape [56].

### Weighted gene co-expression network analysis

Signed co-expression networks were built separately for hiPSC-NPCs and hiPSC-neurons using weighted gene co-expression network analysis (WGCNA) [57]. To construct a global weighted network for each cell type, a total of 15,759 post QC genes across 32 hiPSC-NPCs and 16,721 genes across 42 hiPSC-neurons were used. The absolute values of Pearson’s correlation coefficients were calculated for all possible gene pairs within each cell type and resulting values were transformed using a β-power (β = 12 for hiPSC-NPCs; β = 14 for hiPSC-neurons) so that the final correlation matrices followed an approximate scale-free topology. The WGCNA dynamic tree-cut algorithm was used to detect network modules (minimum module size = 50; cut tree height = 0.99; deep-split = 2, merge module height = 0.20). Once network modules were identified, modules were assessed for significant associations to PMS diagnosis, as well as other biological and technical factors. In order to determine which modules, and corresponding biological processes, were most associated with PMS, we ran singular value decomposition of each module’s expression matrix and used the resulting module eigengene (ME), equivalent to the first principal component, to represent the overall expression profiles for each module. This technique is useful for reducing the number of multiple comparisons from thousands of genes to tens of modules. Gene co-expression modules that were significantly associated with PMS were subjected to functional annotation using the ToppFun module of ToppGene Suite software, as described above. Fisher’s exact tests were used to assess the overlap of co-expression modules between hiPSC-NPCs and hiPSC-neurons, while controlling FDR using the BH procedure.

### Curation of autism and neurodevelopmental disorder gene sets

Two tiers of gene sets were collected to examine overlap with PMS-associated genes in the current study: (1) gene sets implicated in risk for ASD and/or neurodevelopmental disorders (NDDs); and, (2) gene sets that represent differentially expressed genes induced by knockdown (KD) or knockout (KO) of an ASD or NDD gene in hiPSCs. For the genes inplicated in ASD and/or NDDs, we collected loci from (i) five lists of de novo variants implicated in ASD [50, 58–61], (ii) loci that implicate risk for ID [62, 63], and (iii) genes implicated in developmental disorders (DD) from the DDG2P database [64]. We also included genes that are direct targets of FMRP [65]. For the gene sets from other hiPSC transcriptome studies, we curated previously described differentially expressed genes caused by (i) shRNA KD of *SHANK3* in hiPSC-derived neurons [66], (ii) CRISPR/Cas9 heterozygous KO of *CHD8* in hiPSC-derived NPCs and neurons [67], (iii) shRNA KD of *TCF4* and *EHMT1* in hiPSC-derived NPCs [68], (iv) shRNA KD of *MBD5* and *SATB2* in human neural stem cells [69], (v) shRNA KD of *NRXN1* in human neural stem cells [70], and (vi) CRISPR/Cas9 heterozygous and homozygous KO of ten different ASD-related genes in hiPSCs and hiPSC-derived neurons [41]. Full gene lists are provided in Supplemental Table 5.

### Gene overlap analyses

To compute significance of all gene-based overlaps, we used a the GeneOverlap function in R which uses a Fisher’s exact test (FET) and an estimated odds-ratio for all pair-wise tests based on a background set of genes detected in the current study. Overrepresentation of ASD and NDD genetic risk gene sets within gene co-expression modules were also analyzed using a FET to assess the statistical significance. When testing overlap across gene modules, tests were adjusted for multiple testing using BH procedure to control the FDR. Finally, to control for a background set of genes detected in the current data set, this FET function was modified to investigate functional annotations for overlap of differentially expressed genes (DEGs) across independent iPSC studies. Due to the stringency of this test on small-to-medium lists of gene overlaps, enrichment results with *p* value < 0.01 were deemed significant. Notably, many such overlaps survive multiple testing using BH procedure.

## Results

### NPC and Neuron RNA-seq data generation and quality control

**Fig. 1:**
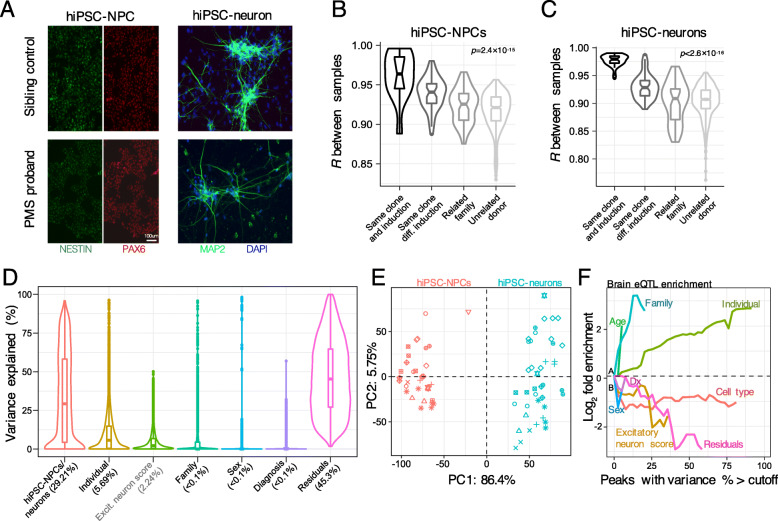
Data quality control metrics. **a** Representative images of hiPSC-NPCs (left) and 6-week-old forebrain neurons (right) from control (top) and PMS probands (bottom). hiPSC-NPCs stained with PAX6 (red), NESTIN (green); hiPSC-neurons stained with MAP2 (green), DAPI-stained nuclei (blue). Pairwise correlations compared (**b**) hiPSC-NPC and (**c**) hiPSC-neuron transcriptomes from the same clone and same induction (*n* = 12, *n* = 31, respectively), same clone but different induction (*n* = 46, *n* = 55, respectively), all related family members (*n* = 68, *n* = 57, respectively) and all unrelated family members (*n* = 505, *n* = 677, respectively). Analysis of variance for multiple comparisons was used to test for differences between the means of correlation coefficients. **d** Linear mixed modelling was used to compute the percentage of gene expression variance explained according to six factors, which represent potential biological sources of variability. Differences in cell types and donor as a repeated measure, followed by excitatory neuron cell composition (estimated using CiberSort in grey) explains the largest amount of variability in the transcriptome data. **e** Principal components analysis of gene expression data from hiPSC-NPCs (red) and hiPSC-neurons (blue), each unique shape denotes one specific donor. Note, there was no distinct stratification by PMS case status based on global expression profiles. (**f**) Genes that vary most across donors are enriched for brain cis-eQTLs. Fold enrichment (log_2_) for the 2000 top cis-eQTLs discovered in post mortem dorsolateral prefrontal cortex data generated by the CommonMind Consortium shown for six sources of variation, plus residuals. Each line indicates the fold enrichment for genes with the fraction of variance explained exceeding the cutoff indicated on the *x*-axis. Enrichments are shown on the *x*-axis until less than 100 genes pass the cutoff

Peripheral blood samples were reprogrammed into hiPSCs and differentiated to generate hiPSC-NPCs from a primary cohort of individuals with PMS (*n* = 7; two males and five females) and their unaffected siblings (*n* = 6; three males and three females; Table 1). Successful differentiation of hiPSC-NPCs into hiPSC-neurons was achieved for five PMS cases and five unaffected siblings (Fig. 1a). The majority of the PMS probands studied here harbor subtelomeric deletions spanning 40–6900 kbp, with the exception of one affected individual with a *SHANK3* point mutation. For all individuals, genome-wide RNA-sequencing was generated from hiPSC-NPCs and from hiPSC-neurons at 4, 6, and 8 weeks in culture, to compare transcriptional differences between PMS probands and their unaffected siblings. For each participant, one to three clones were used for the NPC and neuronal induction yielding a total of 39 hiPSC-NPCs and 41 hiPSC-neurons in the discovery set (Supplemental Table 1). Each hiPSC passed quality control metrics (Supplemental Table 2) while hiPSC-NPCs and hiPSC-neurons were validated using the presence of known cellular markers (Fig. 1a). Subsequently, all gene expression data were inspected for outlier samples on the basis of abnormal gene expression profiles (i.e., samples beyond 95% confidence interval of grand mean), and six hiPSC-NPCs were flagged and removed (Figure S1A-B). Next, because the extent of X-inactivation in females has been reported to be a quality issue during hiPSC reprogramming, we examined the expression patterns of genes on the sex chromosomes using *XIST* on chrX and six genes on chrY for all samples (Figure S1C-D). This analysis identified six female hiPSC-NPCs (five of which were already removed on the basis of outlier expression profiles) that have expression patterns intermediate between males and females, consistent with either contamination or aberrant X-inactivation, which were removed from our analysis. As a general measure, we also confirmed the absence of neural crest cells in our hiPSC-NPCs by querying *AP2* and *SOX10* expression, and both genes went undetected with zero RNA-seq counts. Finally, we queried *SHANK3* gene expression and observed robust, high expression of *SHANK3* in hiPSC-NPCs and hiPSC-neurons in unaffected siblings and marked under-expression in PMS probands (Figure S2).

### Developmental and cellular specificity of hiPSC-NPCs and hiPSC-neurons

We sought to determine whether our hiPSC-NPC and hiPSC-neuronal transcriptome data accurately reflects early developmental gene expression profiles by integrating our RNA-seq data with other studies using hiPSC neuronal cell and postmortem brain gene expression data. A total of 15 independent studies were leveraged covering 11,650 genes and 2719 developmentally distinct samples (see “Materials and methods”). Following standardized data pre-processing procedures, principal component analysis (PCA) stratified all gene expression samples into a distinct developmental axis starting with early embryonic stem cells and subsequently moving into hiPSC-NPCs and hiPSC-neurons, and into prenatal and postnatal postmortem brain samples (Figure S3A). Embryonic stem cells (ESCs) and hiPSCs clustered separately from hiPSC-NPCs and hiPSC-neurons, which in turn co-clustered with early prenatal brain samples. Notably, our hiPSC-NPCs and hiPSC-neurons also co-cluster with hiPSC-NPCs and hiPSC-neurons generated from previous reports, confirming their early developmental gene expression profiles. This clustering was robust to differing methodologies used for hiPSC reprogramming and differentiation across multiple prior studies. We also quantified whether the genes with the highest expression in our hiPSC-NPC and hiPSC-neuron data sets were predominantly prenatally biased in expression using data from the BrainSpan project, and a clear prenatal bias in expression was observed for genes with the highest levels of expression across both cell types (Figure S3B). We also observed that genes with the lowest level of expression were predominantly postnatally biased in expression, indicating that markers of later postnatal brain development are expressed at low levels in the current data sets (Figure S3C).

It is possible that PMS-associated mutations could lead to unique neural cell type composition in proband, as compared to sibling, cells. In addition, genetic background or stochastic factors may also impact cell composition. We therefore estimated proportions of neural cell types for all hiPSC-NPCs and hiPSC-neurons using a reference panel of single-cell RNA-sequencing data from the fetal human cortex [48]. We observed that our hiPSC-NPCs were largely comprised of dividing intermediate progenitor cells (~ 43.4%) and excitatory neurons (~ 23.1%), while hiPSC-neurons were estimated to be comprised predominantly of excitatory neurons (~ 42.4%) and inhibitory neurons (~ 24.8%) (Figure S4). Comparative analyses of the estimated cell type compositions revealed minor increases in predicted proportions of excitatory neuron (*p* = 0.04) and a decrease in inhibitory neurons (*p* = 0.002) in PMS probands relative to unaffected siblings (Figure S4). These in silico predictions suggest that differences in excitatory and inhibitory cell proportions may be impacted by loss of *SHANK3*.

### Quantifying sources of gene expression variability: clinical, technical, and biological factors

Inter-donor and clonal variations have previously been reported to explain a substantial fraction of gene expression variability in hiPSC-derived neural cells. Therefore, as a quality check, genome-wide concordance was evaluated between technical replicates, familial related and unrelated donors. Concordance between technical replicates was examined by either origin of the same clone and the same induction or the same clone but different induction (Fig. 1b, c). Our analysis confirmed that the strongest correlation was observed between technical replicates from the same clone and same induction followed by same clone and different induction in both hiPSC-NPCs and hiPSC-neurons. Subsequently, to test the influence of various factors on gene expression profiles, for each gene, the percentage of gene expression variation attributable to each clinical and technical factor was computed. Collectively, these variables explained ~ 55% of transcriptome variation, with differences between hiPSC-NPCs and hiPSC-neurons having the largest genome-wide effect that explained a median 29.2% of the observed variation, followed by differences in donor as a repeated measure (median 5.2%) and estimated excitatory cell type proportions (median 2.2%) (Fig. 1d). The remaining factors explained smaller fractions of overall transcriptome variation, including family (median < 0.1%) and biological sex (median < 0.1%). Expression variation due to diagnosis (i.e., PMS proband) had a detectable effect in a smaller number of genes. Notably, when hiPSC-NPCs and hiPSC-neurons were analyzed separately, other technical variables such RNA integrity values, sequencing batch, and total number of weeks in culture explained very little expression variation (Figure S5). Additionally, differences in *SHANK3* deletion size had a small but distinct effect on 50 genes, which were significantly overrepresented on chromosome 22 (Figure S6; hiPSC-NPCs, *p* = 1.3e-31; hiPSC-neurons, *p* = 3.2e-18). These genes were encompassed within the largest deletion reported here, and displayed clear patterns of under-expression relative to all unaffected siblings and PMS probands with small deletions (Figure S6 A,D,E).

Next, the influence of cell type proportions, albeit predicted, was further evaluated by overlaying excitatory neuron cell type predictions on a PCA of the gene expression data. The PCA separated hiPSC-NPCs and hiPSC-neurons along the first principal component (PC), explaining 86.4% of the variance, and excitatory neuron cell estimates were separated both by PC1 and PC2 (Fig. 1e). As expected, hiPSC-neurons had a higher proportion of predicted excitatory neurons than hiPSC-NPCs (mean increase = 19.3%, *p* = 1.97e-35 by linear model), and conversely hiPSC-NPCs contain a higher proportion of predicted dividing intermediate neuron progenitor cells (mean increase = 23.7%, *p* = 9.16 = e-63 by linear model), consistent with results derived from our previous analyses. As a final measure, given that a substantial fraction of gene expression variability is explained by inter-donor variation, we asked whether genes with high variance by donor were also enriched for expression quantitative trait loci (eQTLs) detected in postmortem human brain samples. In testing this hypothesis, we confirmed that genes whose variance is largely explained by differences by donor are strongly enriched for brain eQTLs (Fig. 1f), meaning that the observed inter-individual expression variation reflects genetic regulation of expression. However, variation induced by differences in hiPSC-NCPs and hiPSC-neurons, and cell type proportions, did not reflect such genetic differences and it is likely that either stochastic or epigenetic regulators could contribute to their variability.

### Transcriptional signatures of PMS in hiPSC-derived neural cells

**Fig. 2:**
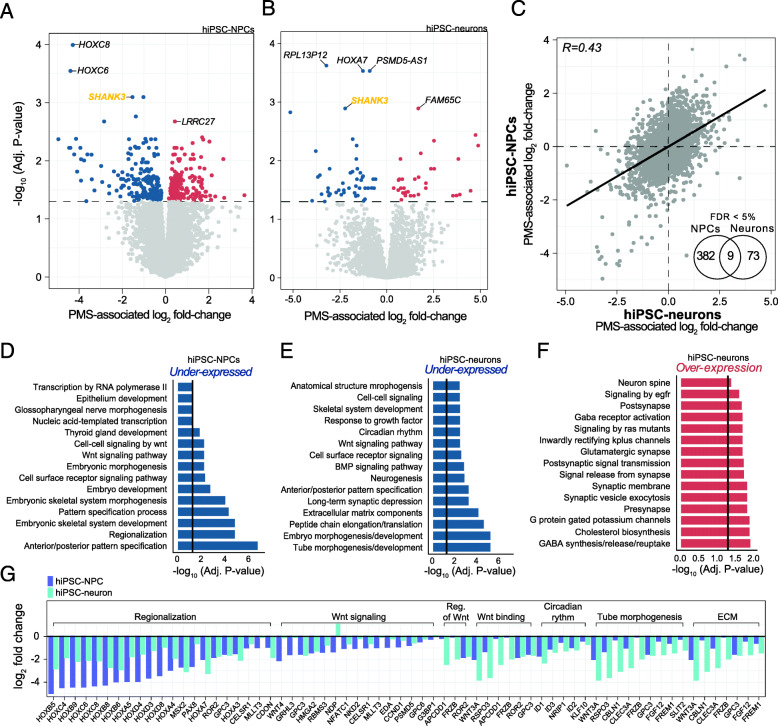
Genes and pathways associated with PMS. Differential gene expression analyses adjusted for sequencing batch, biological sex, RIN, and individual donor as a repeated measure using the dupCorrelation function in the limma R package. Volcano plots compare the extent of PMS-associated log_2_ fold-changes to -log_10_ multiple test corrected *p* value in **a** hiPSC-NPCs and **b** hiPSC-neurons. Black dotted line indicates genes passing an adjusted *p* < 0.05. **c** Genome-wide concordance of PMS-associated log_2_ fold-changes was examined between hiPSC-NPCs and hiPSC-neurons. Inset Venn diagram displays the overlap of significant differentially expressed genes between the two cell types. Functional enrichment analysis of PMS dysregulated genes that show **d** under-expression in hiPSC-NPCs, **e** under-expression in hiPSC-neurons, and **f** over-expression in hiPSC-neurons. All enrichment terms displayed pass a multiple test corrected *p* value. **g** Log_2_ fold-change plot of significantly under-expressed genes in PMS and their respective gene ontology term. *Abbreviations: Reg of Wnt,* regulation of Wnt signaling; *ECM,* extracellular matrix

Differential gene expression analyses comparing PMS probands and unaffected siblings identified 392 differentially expressed genes (DEGs) in hiPSC-NPCs and 82 genes in hiPSC-neurons (FDR < 5%; Fig. 2a, b, Supplemental Table 3), while adjusting for the possible influence of donor as a repeated measure, sex, RIN, and sequencing batch. Genome-wide concordance was examined between hiPSC-NPCs and hiPSC-neurons using PMS-associated log_2_ fold-changes, and a similar patterns of differential gene expression were observed between PMS probands and unaffected siblings in both cell types (Fig. 2c; *R* = 0.43, *p* < 2.2e-16). Moreover, nine statistically significant DEGs were detected across both hiPSC-NPCs and hiPSC-neurons, and each displayed the same direction of effect in PMS, including three genes which were consistently over-expressed (*ARHGAP20*, *PCYT2*, *CAMK2N1*) and six genes which were consistently under-expressed in PMS (*SHANK3*, *PSMD5-AS1*, *GPC3*, *TSHZ2*, *RP11-655M14.13* (*lincRNA*), *RP11-115D19.1* (*lcRNA*)). Functional annotation of DEGs revealed strong pathway and biological enrichment for genes that were predominantly under-expressed in PMS in both hiPSC-NPCs and hiPSC-neurons covering several early developmental terms and pathways, including 20 genes mapping to the Wnt signaling pathway (e.g., *FRZB*, *G3BP1*, *GPC3*, *GPC6*, *MLLT3*, *ROR2*, *RSPO3*, *WNT3A*, *WNT4*) (Fig. 2d, e). Several biological processes were uniquely enriched among the under-expressed genes in hiPSC-neurons, including extracellular matrix (ECM)-related process, BMP signaling, and several broad early developmental terms (e.g., regionalization, embryonic development) (Fig. 2e). We also note the under-expression of circadian rhythm in PMS hiPSC-neurons, including five genes, *NRIP1*, *KLF10*, *ID1*, *ID2*, and *ID3.* Overexpressed genes in hiPSC-neurons also displayed enrichment for genes involved in pre- and postsynaptic activity, cholesterol biosynthesis, transmission across chemical synapses, GABAergic synapses, G protein gated-potassium channels, signaling by insulin receptor, signaling to ERKs, glutamate binding, and activation of AMPA receptors (Fig. 2f). No enrichment was observed for over-expressed genes in PMS hiPSC-NPCs. Notably, adjusting for differing cell type proportions within hiPSC-NPCs and hiPSC-neurons had little effect on the resulting differential gene expression signatures (Figure S7 Supplemental Table 3). A full table of enrichment terms can be found in Supplemental Table 4.

To support these functional enrichment observations, we tested whether candidate genes that are dysregulated together indeed interact with each other at the protein level. A significant overrepresentation of direct protein-protein interactions (PPI) was identified for differentially expressed genes in hiPSC-NPCs (*p* = 2.32e-09, average node degree = 1.81) and hiPSC-neurons (*p* = 4.19e-09, average node degree = 0.81). In hiPSC-NPCs, hub genes in the PPI included genes involved in glutamate receptor signaling pathway, including *GRM3*, *GRIA1*, *CAMK2A*, and several homeobox genes (Figure S8A). The hiPSC-neuron PPI network was notably smaller in edges and nodes, and components of the Wnt signaling pathway emerged as candidate hub genes, including *WNT3A*, *WNT7B*, and *FRZB* (Figure S8B).

Furthermore, we subsetted our RNA-seq data to investigate expression differences in PMS probands with small deletions (*i.e.*, Family ID's 1-5 in Table 1), relative to those observed from a combined analysis of all PMS cases (*i.e.*, Family ID's 1-7 in Table 1). In subsetting PMS cases with small deletions, we identified 44 differentially expressed genes in hiPSC-NPCs and 208 in hiPSC-neurons (Figure S9A-E). Reported genome-wide concordance of log_2_ fold-changes were high when comparing these findings relative to those from the combined analysis of all PMS cases, for both hiPSC-NPCs (*r* = 0.79) and hiPSC-neurons (*r* = 0.90), with strong overlaps for differentially expressed genes. Functional annotation revealed similar biological processes as previously reported (Figure S9 F-G).

As a final measure, given the PMS alterations related to regionalization and Wnt signaling, we examined several region-specific markers (e.g., telecephalic, pallial, subpallial, spinal cord, diencephalic, etc.) to explore whether differences in regional identity induced through passage may be driving the observed differences. As expected, many HOX-related genes, which are commonly of spinal cord and myelencephalic (i.e., posterior) identity, display reduced expression in PMS probands (Figure S10A-B). Nevertheless, when comparing transcriptome-wide patterns from our forebrain hiPSC-neurons with two independent RNA-seq studies, we confirm that our hiPSC-neurons display high transcriptomic similarity with forebrain hiPSC-neurons (median *r* = 0.73) relative to spinal cord motor neurons (median *r* = 0.36), which are more posterior-like (Figure S10C). These results suggest that changes in Wnt signaling, and overlapping regionalization genes, appear to be more of a consequence of *SHANK3* haploinsufficiency than possible differences in underlying regional identities.

### Co-expression modules associated with PMS

**Fig. 3:**
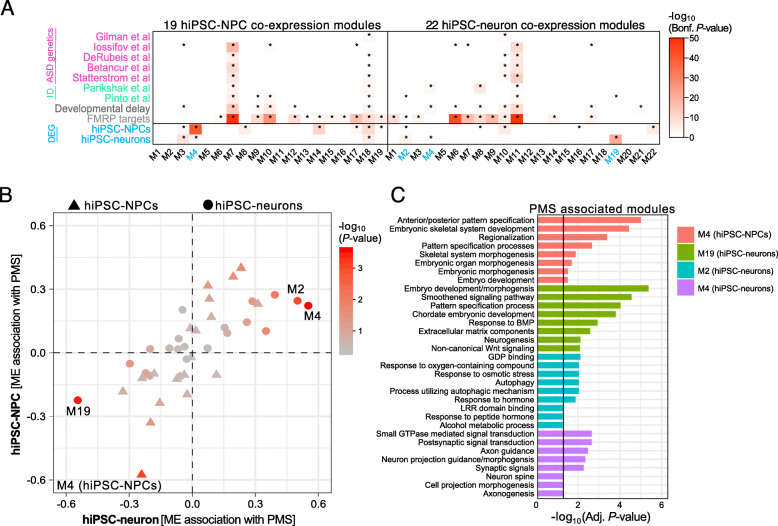
Genes co-expression analysis and enrichment. **a** A total of 19 co-expression modules were identified in hiPSC-NPCs and 22 modules were identified in hiPSC-neurons, and each module was tested for enrichment of genetic risk loci for ASD, ID, and DD using findings from other large-scale studies. Modules were also examined for enrichment of target genes of FMRP, an RNA binding protein that is associated with ASD risk, as well as differentially expressed genes identified in the current study (see Fig. 2). Enrichment was assessed using a Fisher’s exact test to assess the statistical significance and *p* values were adjusted for multiple testing using the Bonferroni procedure. We required an adjusted *p* value < 0.05 (*) to claim that a gene set is enriched within a user-defined list of genes. **b** Module eigengene (ME) values were associated with PMS for hiPSC-NPCs (triangles) and hiPSC-neurons (circles). Next, genes in hiPSC-NPCs were then forced to construct modules using the gene-module assignments identified in hiPSC-neurons, and vice versa, and these ME values were also associated with PMS. **c** Functional enrichment was performed on four PMS-associated modules and the top eight enrichment terms (removing redundant annotations) are displayed

Given that the majority of the PMS-associated genes share similar functions and interactions, we tested whether these genes are also co-expressed. We applied unsupervised WGCNA separately to hiPSC-NPCs and hiPSC-neurons to identify small sets of genes with similar co-expression patterns. A total of 19 co-expression modules were identified in hiPSC-NPCs and 22 modules were identified in hiPSC-neurons, and all modules were well preserved between hiPSC-NPCs and hiPSC-neurons (Figure S11). Each module was assessed for overrepresentation of differentially expressed genes in PMS as well as previously reported genetic risk loci for ASD and other NDDs (Fig. 3a). Genes that were differentially expressed in PMS hiPSC-NPCs were significantly overrepresented in hiPSC-NPC module M4 (∩ = 53, *p* = 1.53e-41), while differentially expressed genes in hiPSC-neurons were strongly enriched across three hiPSC-neuron modules: M2 (∩ = 12, *p* = 0.003), M4 (∩ = 14, *p* = 2.6e-5), and M19 (∩ = 32, *p* = 2.15e-21). Notably, module M2 in hiPSC-neurons harbored a significant fraction of genetic risk loci for ID (∩ = 5, *p* = 0.02), while module M4 in hiPSC-neurons was enriched for DD (∩ = 14, *p* = 0.02) and ID (∩ = 5, *p* = 0.002) risk loci. Module eigengene (ME) values for all modules were then regressed onto individual diagnostic status (i.e., PMS probands), which confirmed significant module-trait associations for module M4 in hiPSC-NPCs with PMS (*r* = − 0.58, *p* = 6e-04) as well as hiPSC-neuron modules M2 (*r* = 0.50, *p* = 9e-04), M4 (*r* = 0.55, *p* = 2e-04), and M19 (*r* = − 0.55, *p* = 2e-04) with PMS (Fig. 3b). Next, the gene-module assignments identified for hiPSC-NPCs and hiPSC-neurons, respectively, were used to perform supervised module construction for the same set of genes in the contrasting cell type (i.e., genes in module M1 identified in hiPSC-NPCs were forced to form a module in hiPSC-neurons), which were similarly tested for association with PMS. In doing so, we found that genes that were either negatively or positively associated with PMS in one cell type, displayed similar levels of association to PMS in the other cell type (Fig. 3b), consistent with our differential gene expression analysis (Fig. 2c). Functional annotation of these candidate modules revealed similar biological functions as previously reported from differential gene expression, including under-expression of hiPSC-NPC module M4 and hiPSC-neuron module M19, which were both enriched for early embryonic development gene sets, ECM, neurogenesis, and Wnt signaling (Fig. 3c). In hiPSC-neurons, module M2 was positively associated with PMS and was implicated in GDP binding, response to oxygen/stress/hormones, LRR domain binding. A separate hiPSC-neuron module M4 was enriched for GTPase signaling, postsynaptic signal transduction, and axon guidance-related processes.

### Overlap with existing ASD transcriptome hiPSC reports

We also explored points of convergence between our *SHANK3*-deficiency findings in PMS with gene expression changes induced by either shRNA knockdown (KD) or CRISPR/Cas9 knockout (KO) of other top ranked ASD genes assayed in neural cell types (Table 2; Figure S12). A total of six hiPSC transcriptome studies spanning 17 different ASD and NDD genes were evaluated. We identified several significant overlaps between PMS-associated gene findings in both hiPSC-NPCs and hiPSC-neurons with gene expression perturbations associated with (i) *SHANK3* KD in neurons [66] (∩ = 20, FET = 2.4e-7; ∩ = 44, FET = 0.003, respectively); (ii) *CHD8* KO in hiPSC-NPCs [67] (∩ = 33, FET = 0.004; ∩ = 15, FET = 0.1.8e-5, respectively); (iii) *CHD8* KO in neurons [67] (∩ = 91, FET = 1.3e-7; ∩ = 29, FET = 5.7e-7, respectively); (iv) *EHMT1* KD in hiPSC-NPCs [68] (∩ = 23, FET = 0.001; ∩ = 10, FET = 0.001, respectively); (v) *NRXN1* KD in stem cells [70] (∩ = 8, FET = 0.001; ∩ = 4, FET = 0.04, respectively); (vi) *SCN2A* KO in hiPSCs [41] (∩ = 55, *p* = 0.0002; ∩ = 16, *p* = 0.001, respectively); (vii) *ATRX* KO in hiPSCs [41] (∩ = 32, FET = 0.03; ∩ = 10, FET = 0.02, respectively); and (viii) *ATRX* KO in neurons [41] (∩ = 47, FET = 6.73e-7; ∩ = 12, FET = 0.002, respectively). In addition, differentially expressed genes in PMS neurons, but not in hiPSC-NPCs, were enriched for genes associated with (ix) *SATB2* KD in stem cells [69] (∩ = 8, FET = 0.0002) and (x) *TNEM1* KO in neurons [41] (∩ = 7, *p* = 0.004). Notably, many of these observed overlaps were enriched for genes implicating changes in Wnt signaling, ECM and perineuronal net (Table 2; Figure S12).

**Table 2:** Overlap of PMS differentially expressed genes (FDR < 5%) with other ASD iPSC transcriptome studies

Study description				hiPSC-NPCs				hiPSC-neurons				Enriched GO terms
Author and year	Target gene	Approach	Cellular model	FET P-value	Odds ratio	Intersect	Example genes	FET P-value	Odds ratio	Intersect	Example genes	
Huang et. 2019	*SHANK3*	shRNA KD	hiPSC-derived neurons	3.3E-04	1.93	44	*COL5A2, COL18A1NDP, ADAMTS18, CRTAP, NFATC1*	2.4E-07	4.64	20	*WNT3A, BCAN, FRZB, SPO3, APCDD1, WNT7B*	Canonical Wnt signaling, perinuclear endoplasmic reticulum lumen
Wang et. 2015	*CHD8*	CRISPR/Cas9 heterozygous KO	hiPSC-derived NPCs	4.0E-03	1.71	33	*CCCND1, VCAN, NFATC1, SMAD9, LRRC8B, HOXB5*	1.8E-05	4.14	15	*FRZB, FREM1, RSPO3, ROR2, MMP15, WNT3A*	Canonical Wnt signaling, golgi lumen, perinuclear endoplasmic reticulum lumen
	*CHD8*	CRISPR/Cas9 heterozygous KO	hiPSC-derived neurons	1.3E-07	1.95	91	*NKD2, COL5A2, COL18A1, NCAN, COL14A1, INHBE*	5.7E-07	3.48	29	*BCAN, FRZB, FREM1, RSPO3, ID1-3, WNT7B*	Non-canonical Wnt signaling pathway, ECM, Glypican pathway
Chen et. 2014	*TCF4*	shRNA KD	hiPSC-derived NPCs	6.2E-01	0.93	5	*ABCB4, FBXL15, SHF, NELL2, ADAMTSL1*	6.8E-01	0.89	1	*PLEKHA5*	NA
	*EHMT1*	shRNA KD	hiPSC-derived NPCs	1.8E-03	2.05	23	*NKD2, CAMK2A, COL18A1, VCAN, NCAN, HOXB6*	1.8E-04	4.52	10	*BCAN, ID2, ID1, WNT7B, ST6GALNAC3, CORO2B*	Golgi lumen, ECM, neuron differentiation
Gigek et al. 2015	*MBD5*	shRNA KD	Human neural stem cells	8.8E-01	0.67	6	*NCAN, SMAD9, E2F7, MARCKSL1, IFITM3, TOP1MT*	5.6E-01	1.07	2	*ID1, KCNJ10*	Perineuronal net, ALK1 signaling
	*SATB2*	shRNA KD	Human neural stem cells	1.7E-01	1.41	11	*NCAN, HMGA2, KCND3, SIGMAR1, DDB2, GAD1*	2.6E-04	5.30	8	*BCAN, ID1, KCNIP1, KLF10, CORO2B, MPC1*	ECM proteoglycans, GABA synthesis, p53 signaling, Ras mediated sigaling
Zeng et al. 2013	*NRXN1*	shRNA KD	Human neural stem cells	1.3E-03	4.06	8	*ZFHX3, NCAN, GFRA1, LRRC8B, DDB2, TMEM151B*	4.2E-02	4.68	4	*HSPA2, NKD2, COL5A2, KCNIP1*	Wnt signaling, golgi lumen, L-glutamate import, ion channel complex
Deneault et al. 2018	*ATRX*	CRISPR/Cas9 heterozygous KO	iPSCs	4.0E-02	1.43	32	*SHANK3, CAMK2N1, INHBE, IGFBP3, SOCS2, GNB4*	2.4E-02	2.20	10	*BCAN, ID2, SHANK3, CAMK2N1, ST6GALNAC3, RHOU*	NA
	*CHD8*	CRISPR/Cas9 heterozygous KO		1.0E+00	0.00	0	*-*	2.4E-01	3.72	1	*OLIG3*	NA
	*AFF2*	CRISPR/Cas9 heterozygous KO		8.8E-01	0.55	2	*CRHBP, GRHL3*	5.4E-01	1.31	1	*HSPA2*	NA
	*CACNA1C*	CRISPR/Cas9 homozygous KO		1.5E-01	1.72	6	*INHBE, GRID2, GPC6, SLC7A2, ADM2, CHMP6*	1.7E-01	2.71	2	*KLF10, ID2*	NA
	*KCNQ2*	CRISPR/Cas9 homozygous KO		6.3E-01	1.01	1	*MDGA2*	1.0E+00	0.00	0	*-*	NA
	*SCN2A*	CRISPR/Cas9 homozygous KO		2.0E-04	2.06	55	*CCND1, NKD2, COL5A2, VCAN, CRTAP, CAMK2A*	1.7E-03	2.56	16	*FRZB, ID1-3, HSPA2, SMOX, ST6GALNAC3, CORO2B*	Voltage gated sodium channel activity, neuronal precursor differentiation
	*ASTN2*	CRISPR/Cas9 homozygous KO		3.3E-01	1.33	5	*INHBE, FOXI3, STK32B, SEMA6D, MANEAL*	5.5E-01	1.25	1	*FOXB1*	Glycoproteins, ECM, Axon guidance
	*DLGAP2*	CRISPR/Cas9 homozygous KO		9.3E-01	0.38	1	*IGFBP3*	1.0E-01	3.76	2	*ST6GALNAC3, RHOU*	NA
	*TENM1*	CRISPR/Cas9 heterozygous KO		1.8E-01	1.40	11	*CAMK2N1, SULT1C4, IGFBP3, CELSR1, MOCOS, FBXO25*	8.2E-01	0.59	1	*CAMK2N1*	NA
	*ANOS1*	CRISPR/Cas9 heterozygous KO		4.0E-01	1.46	2	*MDGA2, SHISA3*	1.0E+00	0.00	0	*-*	NA
	*ATRX*	CRISPR/Cas9 heterozygous KO	iPSC-derived neurons	6.7E-07	2.36	47	*CAMK2A, COL14A1, NCAN, GPC3, SHANK3, TSHZ2*	2.1E-03	2.88	12	*WNT3A, FRZB, GPC3, SHANK3, TSHZ2, ARHGAP20*	Cell migration, positive regulation of Wnt, glutamate receptor activity
	*CHD8*	CRISPR/Cas9 heterozygous KO		1.0E+00	0.00	0		2.7E-02	8.27	2	*PCDHGA3, PCDHGA7*	NA
	*AFF2*	CRISPR/Cas9 heterozygous KO		3.9E-01	1.49	2	*IGFBP3, COL14A1*	3.4E-02	7.27	2	*SLITRK2, PIEZO2*	NA
	*CACNA1C*	CRISPR/Cas9 homozygous KO		6.6E-01	0.94	1	*RASGRP1*	1.0E+00	0.00	0	*-*	NA
	*KCNQ2*	CRISPR/Cas9 homozygous KO		1.0E+00	0.00	0	*-*	1.3E-04	17.19	4	*WNT3A, MSX2, PIEZO2, PCDHGA3*	NA
	*SCN2A*	CRISPR/Cas9 homozygous KO		1.0E+00	0.00	0	*-*	4.2E-02	6.42	2	*PCDHGA3, PCDHGA7*	NA
	*ASTN2*	CRISPR/Cas9 homozygous KO		1.0E+00	0.00	0	*-*	3.3E-05	15.20	5	*WNT3A, ID1, MSX2, PIEZO2, PCDHGA3*	NA
	*DLGAP2*	CRISPR/Cas9 homozygous KO		1.0E+00	0.00	0	*-*	1.0E+00	0.00	0	*-*	NA
	*TENM1*	CRISPR/Cas9 heterozygous KO		2.9E-01	1.23	12	*GPC3, ARHGAP20, COL14A1, VCAN, SLC10A4, CAMSAP3*	5.0E-03	3.60	7	*WNT3A, ID1, MSX2, DPPA4, GPC3, ARHGAP20*	ECM, Glypican pathway, Wnt signaling, signaling patways regulating stem cell pluripotency
	*ANOS1*	CRISPR/Cas9 heterozygous KO		1.0E+00	0.00	0	*-*	6.6E-03	17.77	2	*WNT3A, PCDHGA3*	NA

A Fisher’s exact test (FET) and an estimated odds-ratio were used to compute significance of each overlap. When the intersection is greater than six, only six intersecting example genes are displayed for brevity. All overlapping genes found in common with the current study were pooled and subjected to pathway analyses using FET and a genome background set to 17353 genes

*KD* knockdown, *KO* knockout

### Validation of PMS-associated gene dysregulation in hiPSC-neurons

**Fig. 4:**
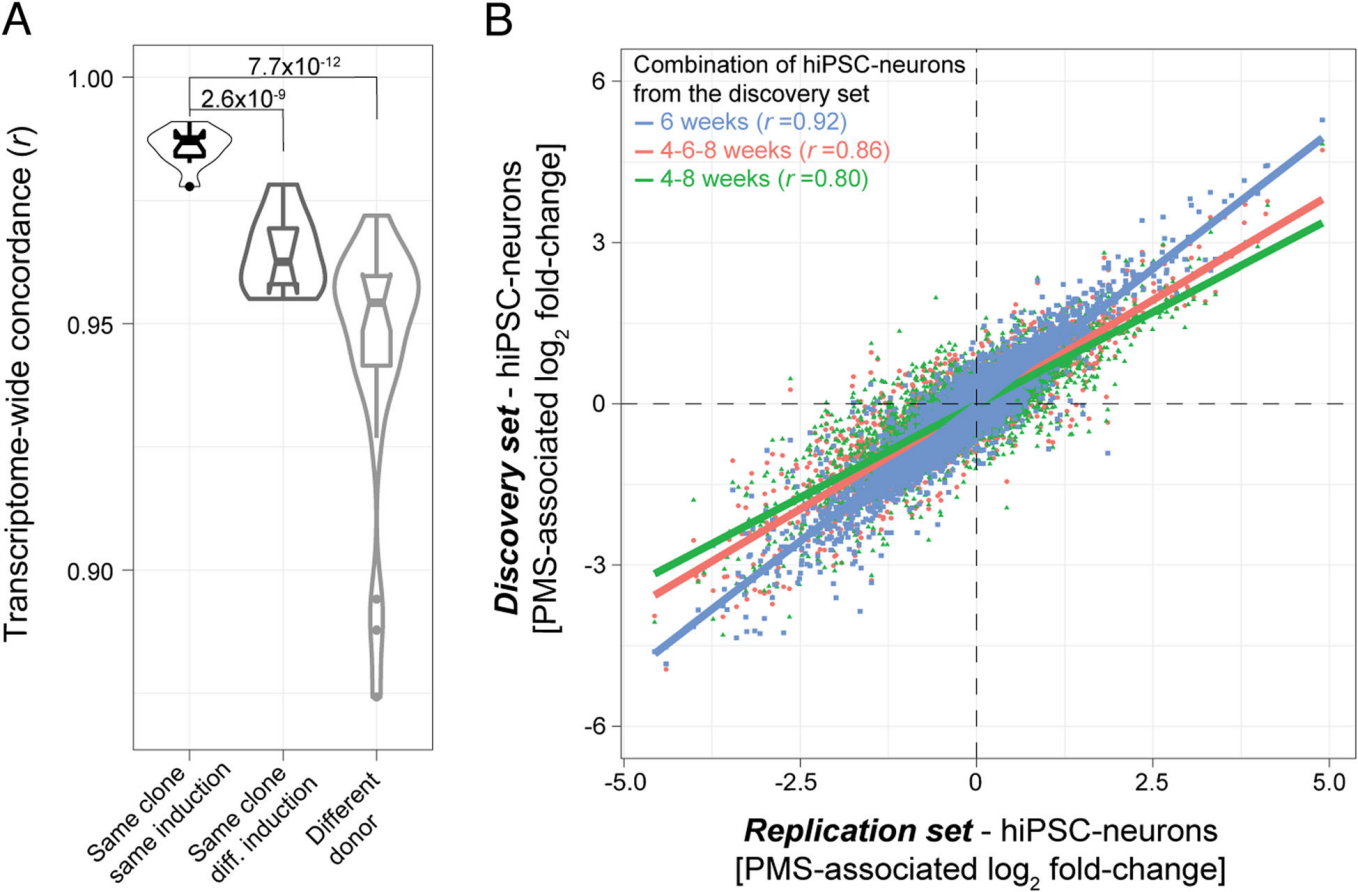
Replication of hiPSC-neuron RNA-seq. A replication set of hiPSC-neurons collected at 6 weeks in culture were subjected to RNA-seq. **a** Correlation coefficients between samples from the same donor and same clone (technical replicates), same clone but different induction (biological replicates), and correlations between all other samples. A Wilcoxon rank-sum test was used to test for differences between the means of correlation coefficients. **b** The second replication batch of hiPSC-neurons were used to derive differential gene expression signatures between PMS probands and unaffected siblings. The PMS-associated log_2_ fold-changes from this replication set (*x*-axis) were compared to PMS-associated log_2_ fold-changes from the discovery set of samples, which were derived using combinations technical replicates and biological replicates at different weeks in culture (*y*-axis)

To validate our PMS transcriptional signatures, we performed additional RNA-sequencing on a replication set of 21 hiPSC-neurons collected at 6 weeks. Following data preprocessing, four samples were removed on the basis of aberrant X-inactivation (Figure S13A-B) and a total of eight biological replicates derived from independent differentiations and nine technical replicates passed into our subsequent validation analyses. In silico predictions of cell type frequencies validated the trending decreases in inhibitory neurons (*p* = 0.05) and increases in excitatory neurons (*p* = 0.08), albeit to an insignificant extent in PMS (Figure S13C). Subsequently, sample-to-sample correlation coefficients were evaluated between discovery and replication samples, first among technical replicates (median *r* = 0.98), then among biological replicates derived from independent differentiations (median *r* = 0.96) and between unrelated donors (median *r* = 0.94) (Fig. 4a). Overall, levels of concordance were highest between technical replicates relative to those observed between biological replicates (*p* = 2.6e-9) and unrelated donors (*p* = 7.7e-12). Next, differentially expressed genes were computed using the replication set of neurons and genome-wide concordance of PMS-associated log_2_ fold-changes were regressed onto log_2_ fold-changes computed using different combinations of discovery set hiPSC-neurons: (i) 6-week samples; (ii) 4- and 8-week samples; or (iii) 4-, 6-, and 8-week samples. As expected, the highest levels of concordance were observed between discovery and replication 6-week samples (*r* = 0.92) followed by a combination of 4, 6, and 8 weeks (*r* = 0.86) and subsequently 4- and 8-week samples (*r* = 0.80) (Fig. 4b). Moreover, using this replication set, we observed similar under-expression of PMS-related gene sets implicated broad early developmental pathways as well as ECM-related terms and Wnt-signaling (Supplemental Table 4).

## Discussion

Our study sought to characterize transcriptional signatures of *SHANK3* haploinsufficiency in neurodevelopment, by comparing genome-wide RNA-seq profiles of hiPSC-NPCs and hiPSC-neurons derived from individuals with PMS with those of their unaffected siblings. We report on the largest sample set of PMS-derived hiPSC-NPCs and hiPSC-neurons to date, representative of a range of genetic lesions associated with PMS, from a *SHANK3* point mutation to small and large 22q13.3 deletions. DEGs in our dataset were enriched for pathways involved in core developmental processes such as pattern specification and embryonic morphogenesis, including Wnt signaling pathways that are essential for neuronal fate specification. Gene co-expression modules generated from these data demonstrated convergence between altered PMS molecular pathways and ASD and NDD genetic risk loci. Importantly, overlapping DEG findings were identified between the current study and findings from other ASD and NDD transcriptome hiPSC studies, demonstrating overlapping changes in RNA involved in Wnt signaling and in the ECM.

The transcriptional signatures of PMS in hiPSC-neurons point to altered postsynaptic density and membrane, perisynaptic extracellular matrix, synaptic signaling, and organization and GABAergic genes. Our results are in line with evidence supporting a role for *SHANK3* prior to synaptogenesis and neural circuit formation, specifically in early morphogenesis and neurogenesis [32, 65, 71–74]. For example, a zebrafish model of PMS that utilized morpholinos to disrupt shank3a and shank3b resulted in delayed mid- and hindbrain development, with associated disruptions in motor behaviors, and the emergence of seizure-like behaviors [73].

*SHANK3* may mediate presynaptic function via transsynaptic signaling through cell adhesion molecules such as neurexin and neuroligin [75, 76]. In a rat hippocampal in vitro model, *SHANK3* expression was found to affect transsynaptic signaling by modulating pre- and postsynaptic protein content and neurotransmission efficiency through neurexin-neuroligin interactions [74]. *SHANK3* has been shown to bind neuroligin via its PDZ domain [77], therefore potentially regulating synaptic strength via retrograde signaling through cell adhesion molecules. In addition, since neurexin-neuroligin are implicated in the regulation and coordination of synaptic function via transsynaptic signaling [76], with some evidence of NMDAR regulation involvement [78], their association with *SHANK3* presents with one possible mechanism by which *SHANK3* disruption could dysregulate E/I balance in the developing brain. Moreover, our results suggest that disrupted neurogenesis in PMS hiPSC-NPCs may precede changes in hiPSC-neurons and, in part, alter the ratio of distinct neuronal subtypes, with possible consequences on E/I balance. Complimentary to this notion, previous hiPSC work in ASD has identified early neural progenitor stages as critical periods that may contribute to future disease propensity, and notably report gene expression changes associated with cell cycle and overproduction of GABAergic neurons [40].

Our group has previously shown that *SHANK3* point mutations are sufficient to convey a PMS phenotype [4], although larger deletion sizes have been associated with a more severe range of PMS manifestations [6, 7, 41]. Here, we find that differences in *SHANK3* deletion size have a significant dosage effect on 50 genes that span the largest deletion in our dataset. One of these genes, *WNT7B*, had been previously associated with macrocephaly and chromosome 22 deletions greater than 5Mb [7]. *WNT7B* codes for a secreted signaling protein that is central to Wnt signaling pathway, which is also enriched in our dataset and has been implicated in *SHANK3* deficiency in previous reports [79]. These findings suggest that genes in close proximity to *SHANK3* on chromosome 22 may also play a role in modulating the pathobiology of PMS, particularly in individuals with deletions.

We also note downregulation of *HOX* genes in PMS hiPSC-NPCs and hiPSC-neurons, which is consistent with our reported deficits in patterning and maturity. While HOX genes are critical for brain patterning in the development of the hindbrain [80], they are also important in the forebrain (see review, [81]), and abnormal expression has been implicated in ID, subtypes of ASD, and epilepsy [82–84].

Furthermore, we identified several points of convergence on ECM and Wnt signaling in PMS and other hiPSC studies of ASD and NDDs. Numerous lines of evidence point to Wnt signaling as a candidate pathway implicated in ASD etiology [85]. In previous reports, mutations of Wnt signaling pathway genes involved in processes such as neurite growth, synapse formation, neurogenesis, and corticogenesis have been associated with ASD phenotypes [86, 87]. For example, *CTNNB1*, which produces the protein β-catenin, plays a critical role in cell adhesion and cell signaling in the Wnt signaling pathway and de novo mutations in *CTNNB1* have been linked to individuals with DD, ID, and ASD [50]. In murine models, stabilization of *CTNNB1* in cortical samples has been found to increase Wnt signaling and boost neurogenesis [88], while depletion of *CTNNB1* from inhibitory neurons leads to deficits in neuronal activation and ASD-like behavior [89]. Numerous top-ranked ASD risk genes have also been found to function with *CTNNB1* and the Wnt signaling pathway. For example, *CHD8* is a chromatin remodeling factor and a top-ranked ASD risk gene, which has been shown to be a positive regulator of *CTNNB1*-mediated Wnt signaling in hiPSC-NPCs [90]. Notably, many under-expressed genes in the current dataset show enrichment for both Wnt signaling genes and *CHD8* binding sites. Both *PTEN* and *TCF7L2* also represent ASD and ID risk genes [58, 60, 91, 92], respectively, and have been identified to function with *CTNNB1* to regulate normal brain growth [93] and to initiate transcriptional responses following Wnt receptor binding [94]. Additionally, de novo mutations in *DDX3X* are associated with ID and ASD [95], and this gene has been recently identified to be an important component of *CTNNB1*-mediated Wnt signaling by regulating kinase activity, which in turn promote phosphorylation of Dvl and represents a major hub in the Wnt pathway [96]. Therefore, in addition to ASD, *CTNNB1*-mediated Wnt signaling may be disrupted in ID and other NDDs, further underscoring the points of convergence identified the current study and demonstrating the importance of this pathway in proper neurodevelopment.

Given the described changes in the Wnt signaling pathway, a follow-up question would be whether pharmacological regulation of Wnt signaling represents an important and/or plausible treatment strategy for PMS and ASD. Notably, several medications have been shown to modulate Wnt signaling, including methylphenidates [97], selective serotonin reuptake inhibitors (SSRIs) [98], and some antipsychotic medications [94, 99]. For example, long-term administration of methylphenidate in mice has been shown to modulate key components of the Wnt signaling pathway, including Akt and GSK3 [97]*.* Similarly, SSRI treatment (e.g., fluoxetine) has been shown to boost Wnt signaling, specifically *Wnt2* and *Wnt3,* in two different mouse studies [98, 100]. Furthermore, two additional reports have also demonstrated that the antipsychotic medication haloperidol promotes Wnt signaling, including modulating *WNT5A* and β-catenin expression [101] as well as the phosphorylation of Akt [99]. Overall, it is noteworthy that several pharmacological interventions for behavioral disorders affect components of Wnt signaling, either directly or indirectly. However, additional investigations are required to determine the role of these mechanisms and compare the treatment efficacy across individuals with and without Wnt signaling abnormalities.

The current study also presents some limitations. First, given the rarity of PMS, we could not carry out experimental validation in independent biological samples. Nevertheless, in an effort to boost signal over noise, several points of convergence were identified with gene-based findings from other hiPSC transcriptome studies. Second, loss of *SHANK3* has been shown in numerous prior reports to affect neurite length, complexity of neurite arborization, and soma area [31–36], which were not examined in the current study and may contribute to some of the observed transcriptional changes. Third, while in silico predictions of cell type proportions attempted to control and quantify the variance in these transcriptome data, it remains possible that some transcriptional changes can be related to changes in proportions of specific cell types. This is especially true when studying neurons, which reflect a heterogeneous mixture of neuronal subpopulations and mixed glial cells. Single-cell RNA-sequencing of neurons at different differentiation stages may produce a clearer picture of the underlying cellular heterogeneity and corresponding gene expression profiles in such samples. Finally, it is worth noting that while the current study examined forebrain hiPSC-NPCs, hiPSC-NPCs can drift to become more posterior-like with passaging [102], and perhaps underlying differences in regional identities could drive the observed alterations in Wnt signaling and HOX genes. To maintain low regional heterogeneity, we used only low passage NPCs (maximum of 6) which have less propensity to spontaneously differentiate into neurons. And, while we find our hiPSC-neurons show more forebrain than posterior identity, future work may compare and contrast gene expression and cellular phenotypes of PMS-derived hiPSCs that are differentiated to reflect distinct regional identities.

## Conclusion

In summary, our study demonstrates that *SHANK3*-deficiency results in significant transcriptional changes in PMS-derived hiPSC-NPCs and hiPSC-neurons. Many early developmental pathways are impacted, including altered pre- and postsynaptic signaling, embryonic development and function, as well as Wnt and ECM signaling. Several other hiPSC transcriptome studies of ASD and NDD genes also displayed changes in ECM and Wnt signaling, providing molecular insights into PMS and into NDDs more broadly.

## Supplementary information

**Additional file 1: Figure S1.** RNA-seq quality control. Principal component analyses were performed on RPKM values for all (A) hiPSC-NPC and (B) hiPSC-neuron gene expression samples. Outliers beyond the 95% confidence intervals (black ellipse) were excluded from downstream analyses. We also sought to identify samples that may have under-gone issues with X-inactivation and/or sample mislabeling by confirming that the reported biological sex is concordant with gene expression on chrX and chrY for both (C) hiPSC-NPCs and (D) hiPSC-neurons. The expression on XIST from chrX was plotted against the sum of expression of six chrY genes (*USP9Y*, *UTY*, *NLGN4Y*, *ZFY*, *RPS4Y1*, *TXLNG2P*). Female samples with intermediate expression profiles were excluded from further analysis.

**Additional file 2: Figure S2.** SHANK3 gene expression. (A) *SHANK3* gene expression (RPKM) across hiPSC-NPCs and hiPSC-neurons. (B) *SHANK3* read coverage in other hiPSC-NPC studies, demonstrating *SHANK3* is expressed in hiPSC-NPCs. (C) *SHANK3* transcript expression across PMS probands and sibings for hiPSC-NPC and hiPSC-neuronal samples. Analysis of variance was used to test for *SHANK3* transcript expression differences between PMS probands and unaffected siblings (SHANK3 ~ Diagnosis) as well as the interaction between time and diagnosis (SHANK3 ~ Time point + Diagnosis).

**Additional file 3: Figure S3.** Developmental specificity analysis. (A) Several postmortem brain and hiPSC RNA-seq data sets spanning a broad range of developmentally distinct samples were integrated with the hiPSC-derived hiPSC-NPCs and hiPSC-neurons in the current study by principal component analysis to confirm their developmental specificity. The first two principal components are shown and the hiPSC-NPCs (black stars) and hiPSC-neurons (black triangles) are each outlined by 95% confidence intervals. A t-statistic was calculated comparing prenatal to postnatal expression in the BrainSpan bulk RNA-seq data. (B) In hiPSC-NPCs, the t-statistic distribution of the top 1000 most expressed shows a prenatal bias and the top 1000 least expressed genes shows a clear postnatal bias. (C) A similar pattern was observed for the top 1000 most and least expressed genes across hiPSC-neurons.

**Additional file 4: Figure S4.** Cell type deconvolution analysis. Cibersort cell type deconvolution analysis of global gene expression profiles estimated cell frequencies (y-axis) in (A-B) hiPSC-NPCs and (C-D) hiPSC-neurons for four major cell types (x-axis) using a reference panel of single-cell RNA-sequencing data from the human fetal cortex. The predicted cellular proportions were compared between PMS probands and unaffected siblings to confirm that major shifts in underlying cell types would not confound downstream analyses. A Wilcox rank-sum test was used to compare the fractions of cell proportions between probands and siblings.

**Additional file 5: Figure S5.** Variance explained by technical factors. The linear mixed model framework of the varianceParition R package was used to compute the percentage of gene expression variance explained by multiple biological and technical factors for (A) hiPSC-NPCs and (B) hiPSC-neurons. (C) The variance explained by the total number of weeks hiPSC-neurons spent in culture was further evaluated by principal component analysis, and each unique shape reflects one specific donor.

**Additional file 6: Figure S6.** Variance explained by SHANK3 deletion size. (A) All genes affected by chr22 deletion in PMS proband from family 6 (4.9Mb deletion) are similarly affected in PMS proband from family 7 (6.9Mb deletion). (B) The linear mixed model framework of the varianceParition R package was used to compute the percentage of gene expression variance explained by *SHANK3* deletion size in hiPSC-NPCs and hiPSC-neurons. (C) Genes with variance explained >50% by deletion size were examined for chromosomal enrichment, and strong enrichment for chromosome 22 was observed. The vertical black line indicates -log_10_ P-value < 0.05. Fifty unique genes were identified that varied by deletion size and mapped to chromosome 22, which were plotted on a heatmap using average expression values across all technical replicates for (D) hiPSC-NPCs and (E) hiPSC-neuronal samples. *SHANK3* deletion sizes are displayed on the x-axis, and correspond to those present in Table 1.

**Additional file 7: Figure S7.** GO semantic similarity and incorporating cell type frequencies for differential expression. GO semantic similarity analysis was applied to examine shared/unique gene content among significantly under-expressed GO terms in (A) hiPSC-NPCs and (B) hiPSC-neurons. GO terms were then clustered based on ward and Euclidean distance and Ward’s clustering. The concordance of genome-wide PMS-associated log_2_ fold-changes were evaluated comparing two models: i) one model adjusting for sequencing batch, biological sex, RIN and individual donor as a repeated measure on the y-axis; and ii) a second model adjusting for the same factors plus predicted excitatory neuron cell type composition on the x-axis. Concordance was examined for both (C) hiPSC-NPCs and (D) hiPSC-neurons.

**Additional file 8: Figure S8.** Protein-protein interaction network. Direct protein–protein interaction network of differentially expressed genes identified in (A) hiPSC-NPCs and (B) hiPSC-neurons. Nodes are scaled by their degree of overall connectivity in the network.

**Additional file 9: Figure S9.** Differential expression in small deletion PMS cases. Genome-wide concordance of log_2_ fold-changes were examined for small deletion cases using (A) hiPSC-NPCs (4 PMS cases and 4 unaffected siblings, y-axis) and (B) hiPSC-neurons (3 PMS cases and 3 unaffected siblings, y-axis) relative to a pooled sample analysis as described in Fig. 2 (x-axes, respectively). Overlap of differentially expressed genes detected (C) in hiPSC-NPC small deletion cases compared to the pooled analysis (D) in hiPSC-neuron small deletion cases compared to the pooled analysis, and (E) between small deletion cases in hiPSC-NPCs and hiPSC-neurons. Gene ontology analysis of under-expressed genes in the PMS cases with small deletions were reported for (F) hiPSC-NPCs and (G) hiPSC-neurons.

**Additional file 10: Figure S10.** Regional marker genes. Marker genes covering eight different regional identities were evaluated for (A) hiPSC-NPCs and (B) hiPSC-neurons. Averaged expression values across 1-3 clones per donor for each marker were used to generate a heatmap. Regional identities (left of heatmap) and gene symbols (right of heatmap) are displayed. Heatmap colors are scaled from low (blue) medium (white) and high (red) expression. (C) Transcriptome-wide Pearson’s correlation between our hiPSC-neurons relative to two independent RNA-seq studies on forebrain hiPSC-neurons and spinal cord motor neurons, which are more posterior-like.

**Additional file 11: Figure S11.** WGCNA module construction and overlap. The β-power defined for both (A) hiPSC-NPCsand (B) hiPSC-neurons in order to achieve scale free network topology for gene co-expression network construction. As a rule of thumb, β-power’s > 0.8 achieve scale free network topology, and a final β-power of 12 was used hiPSC-NPCs and a β-power of 14 for hiPSC-neurons. (C) Overlap analysis of co-expression modules defined based on hiPSC-NPCs and hiPSC-neurons. Significance of the overlap was tested using a one-sided Fisher’s exact test and corrected for multiple comparisons using Bonferroni procedure. Significant overlaps (*) are reported for overlaps displaying adjusted *P*<0.05.

**Additional file 12: Figure S12.** Overlap with other ASD hiPSC transcriptome studies. Convergence of differentially expressed genes (FDR <5%) in the current study with other ASD hiPSC transcriptome studies was assessed using a Fisher’s Exact Test (FET) and an estimated odds-ratio was computed in comparison to a genome-wide background set to 20,000. Significant overlaps are demarked with a red asterisks (*). All overlapping genes found in common with the current study were pooled and subjected to functional enrichment using FET and adjusting for genome background of 17,353 genes. Overlap of differentially expressed genes and functional annotation was performed using data from (A-B) Huang et al., 2019, (C-D) Wang et al., 2015, and (E-G) Chen et al., 2014, (H) Gigek et al., 2015, (I-J) Zheng et al., 2013, and (K-M) Deneault et al., 2018. Note that no functional enrichment was observed based on the overlap with (G) Chen et al., 2014 and (H) Gigek et al., 2015. To simply multiple overlaps, (K-L) the -log_10_ P-value (x-axis) is used to display the extent of significance based on gene expression perturbations associated with CRISPR/Cas9 knockout of 10 different ASD genes.

**Additional file 13: Figure S13.** Data pre-processing using replication hiPSC-neurons. Principal component analyses were performed on RPKM values for (A) all replication set hiPSC-neurons at 6 weeks. Outliers beyond the 95% confidence intervals (black ellipse) were excluded from downstream analyses. (B) We also sought to identify samples that may have under-gone issues with X-inactivation and/or sample mislabeling by confirming that the reported biological sex is concordant with gene expression on chrX and chrY, which confirmed aberrant X-inactivation observed in hiPSC-NPCs sharing the same clone and induction (Supplemental Table 1). Samples with intermediate expression profiles were excluded from further analysis. (C) Cibersort cell type deconvolution analysis of global gene expression profiles estimated cell frequencies (y-axis) for four major cell types (x-axis) using a reference panel of single-cell RNA-sequencing data from the human fetal cortex. The predicted cellular proportions were compared between PMS probands and unaffected siblings using a Wilcox rank-sum test.

**Additional file 14: Table S1.** Summary of the total number of clones and inductions for each sample.

**Additional file 15: Table S2.** The hiPSC Scorecard assay is a qPCR-based panel of marker genes for pluripotency.

**Additional file 16: Table S3.** Differentially expressed genes between PMS probands and unaffected siblings in hiPSC-NPCs and hiPSC-neurons.

**Additional file 17: Table S4.** Pathway and functional annotation enrichment results.

**Additional file 18: Table S5.** Manually curated gene sets used to assess overlap with findings from the current study.

## Abbreviations

hiPSCs: Human induced pluripotent stem cells
PMS: Phelan-McDermid syndrome
ASD: Autism spectrum disorder
NDD: Neurodevelopmental disorder
ID: Intellectual disability
RNA-seq: RNA-sequencing
NPCs: hiPSC-derived neural progenitor cells
Neurons: hiPSC-derived neurons
DEG: Differentially expressed gene
KD: Knockdown
KO: Knockout
ECM: Extracellular matrix
