# Supplementary figures and images for: Transcriptional signatures of participant-derived neural progenitor cells and neurons implicate altered Wnt signaling in Phelan-McDermid syndrome and autism
Source: Mol Autism. 2020 Jun 19;11:53. doi: 10.1186/s13229-020-00355-0 (PMC7304190; doi:10.1186/s13229-020-00355-0)

A

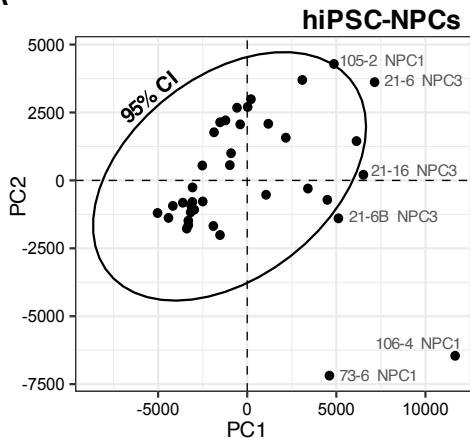

B

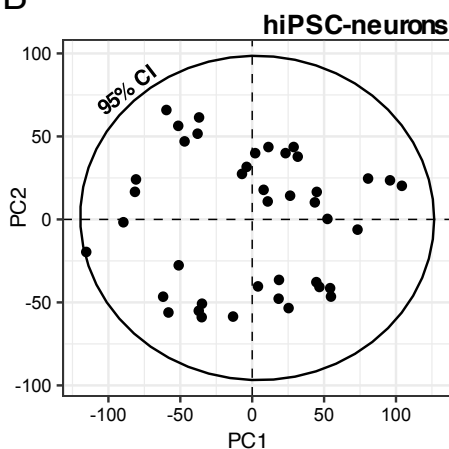

C

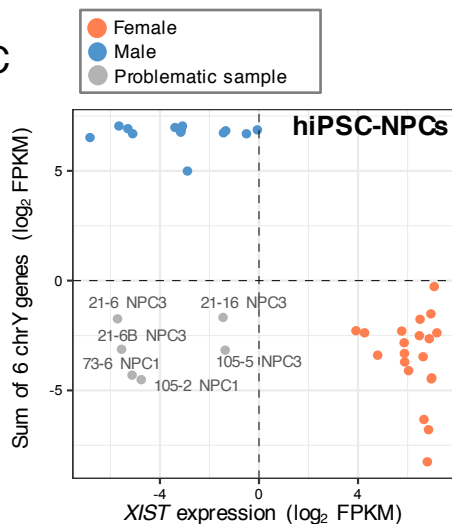

D

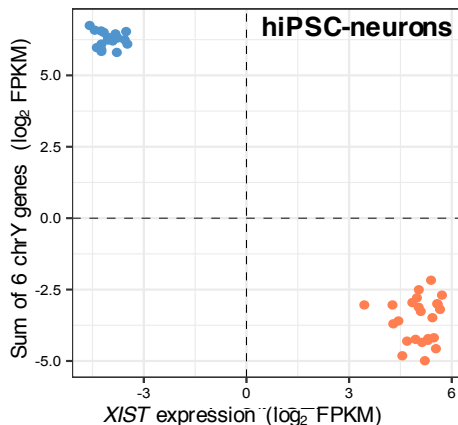

Supplement: Supplementary file 1 — Additional file 1: Figure S1. RNA-seq quality control. Principal component analyses were performed on RPKM values for all (A) hiPSC-NPC and (B) hiPSC-neuron gene expression samples. Outliers beyond the 95% confidence intervals (black ellipse) were excluded from downstream analyses. We also sought to identify samples that may have under-gone issues with X-inactivation and/or sample mislabeling by confirming that the reported biological sex is concordant with gene expression on chrX and chrY for both (C) hiPSC-NPCs and (D) hiPSC-neurons. The expression on XIST from chrX was plotted against the sum of expression of six chrY genes (USP9Y, UTY, NLGN4Y, ZFY, RPS4Y1, TXLNG2P). Female samples with intermediate expression profiles were excluded from further analysis. [file 13229_2020_355_MOESM1_ESM.pdf]

A

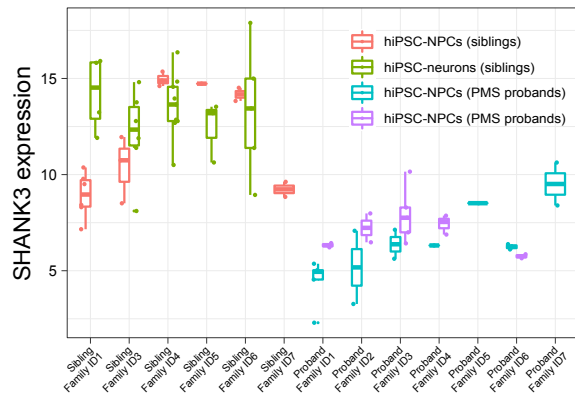

B

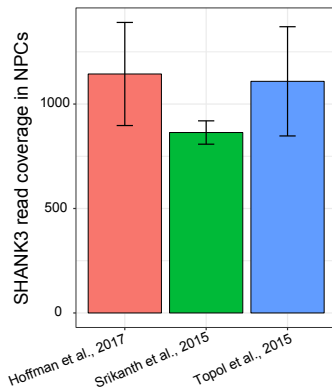

C

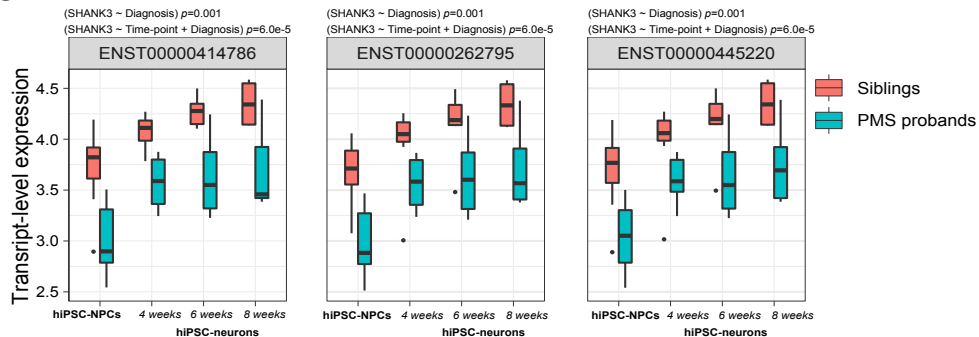

Supplement: Supplementary file 2 — Additional file 2: Figure S2. SHANK3 gene expression. (A) SHANK3 gene expression (RPKM) across hiPSC-NPCs and hiPSC-neurons. (B) SHANK3 read coverage in other hiPSC-NPC studies, demonstrating SHANK3 is expressed in hiPSC-NPCs. (C) SHANK3 transcript expression across PMS probands and sibings for hiPSC-NPC and hiPSC-neuronal samples. Analysis of variance was used to test for SHANK3 transcript expression differences between PMS probands and unaffected siblings (SHANK3 ~ Diagnosis) as well as the interaction between time and diagnosis (SHANK3 ~ Time point + Diagnosis). [file 13229_2020_355_MOESM2_ESM.pdf]

A

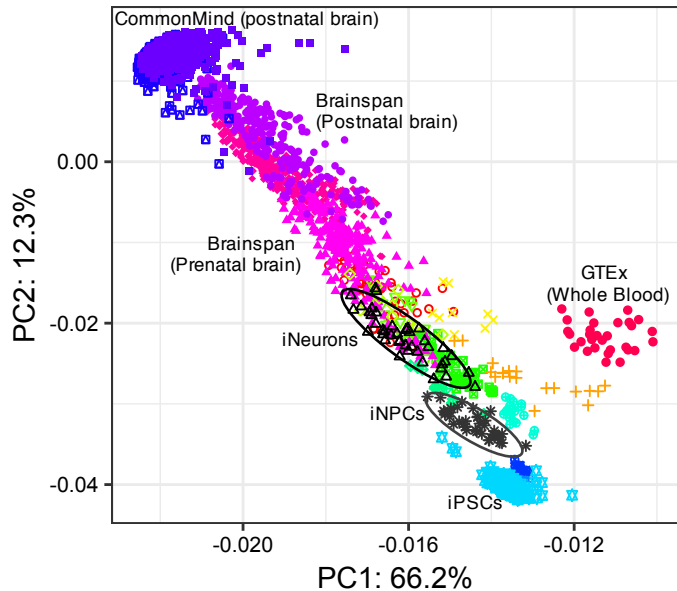

B

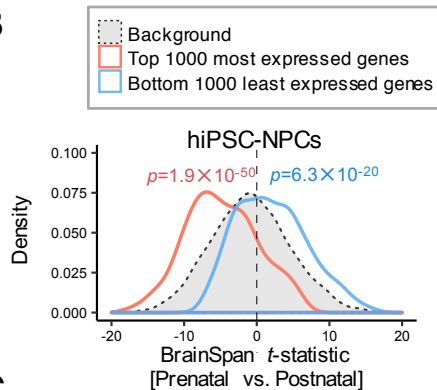

C

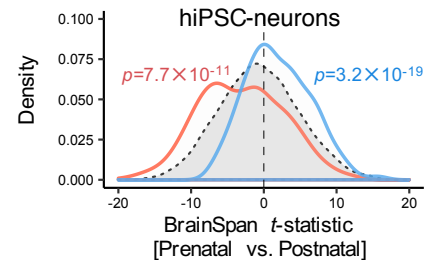

Supplement: Supplementary file 3 — Additional file 3: Figure S3. Developmental specificity analysis. (A) Several postmortem brain and hiPSC RNA-seq data sets spanning a broad range of developmentally distinct samples were integrated with the hiPSC-derived hiPSC-NPCs and hiPSC-neurons in the current study by principal component analysis to confirm their developmental specificity. The first two principal components are shown and the hiPSC-NPCs (black stars) and hiPSC-neurons (black triangles) are each outlined by 95% confidence intervals. A t-statistic was calculated comparing prenatal to postnatal expression in the BrainSpan bulk RNA-seq data. (B) In hiPSC-NPCs, the t-statistic distribution of the top 1000 most expressed shows a prenatal bias and the top 1000 least expressed genes shows a clear postnatal bias. (C) A similar pattern was observed for the top 1000 most and least expressed genes across hiPSC-neurons. [file 13229_2020_355_MOESM3_ESM.pdf]

**A**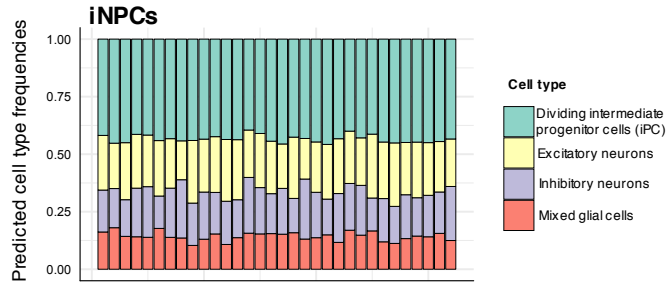**B**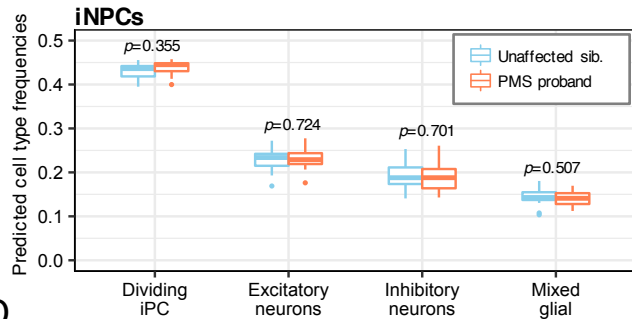**C**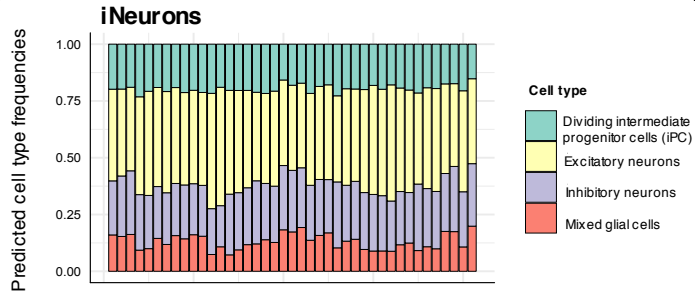**D**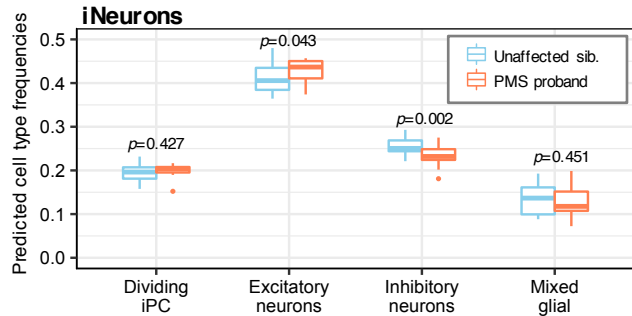

Supplement: Supplementary file 4 — Additional file 4: Figure S4. Cell type deconvolution analysis. Cibersort cell type deconvolution analysis of global gene expression profiles estimated cell frequencies (y-axis) in (A-B) hiPSC-NPCs and (C-D) hiPSC-neurons for four major cell types (x-axis) using a reference panel of single-cell RNA-sequencing data from the human fetal cortex. The predicted cellular proportions were compared between PMS probands and unaffected siblings to confirm that major shifts in underlying cell types would not confound downstream analyses. A Wilcox rank-sum test was used to compare the fractions of cell proportions between probands and siblings. [file 13229_2020_355_MOESM4_ESM.pdf]

A

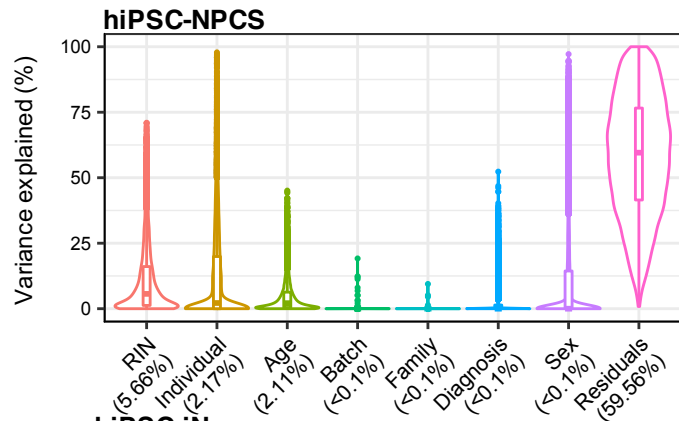

B

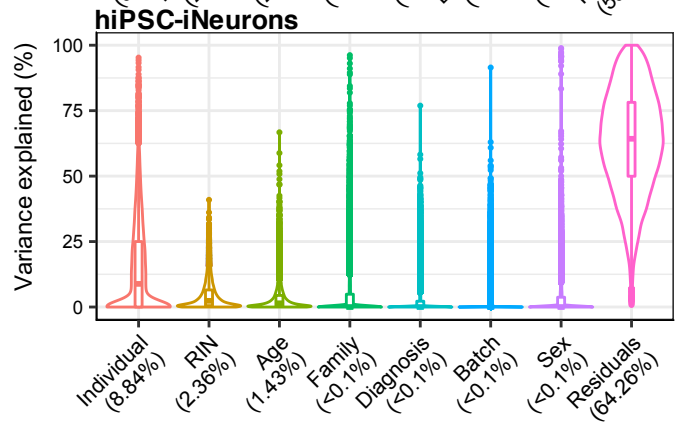

C

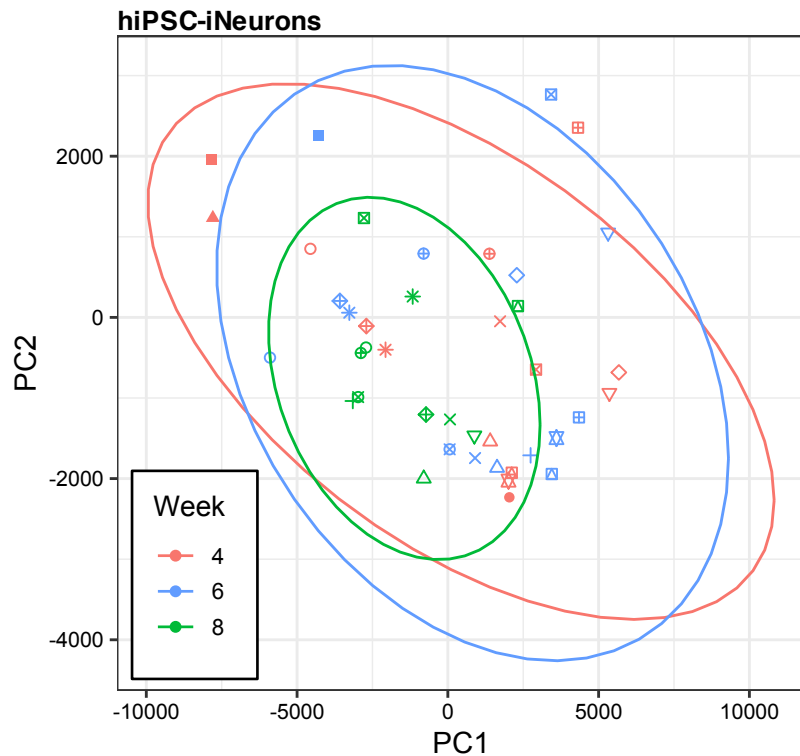

Supplement: Supplementary file 5 — Additional file 5: Figure S5. Variance explained by technical factors. The linear mixed model framework of the varianceParition R package was used to compute the percentage of gene expression variance explained by multiple biological and technical factors for (A) hiPSC-NPCs and (B) hiPSC-neurons. (C) The variance explained by the total number of weeks hiPSC-neurons spent in culture was further evaluated by principal component analysis, and each unique shape reflects one specific donor. [file 13229_2020_355_MOESM5_ESM.pdf]

A

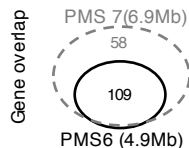

B

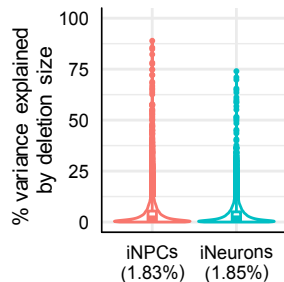

C

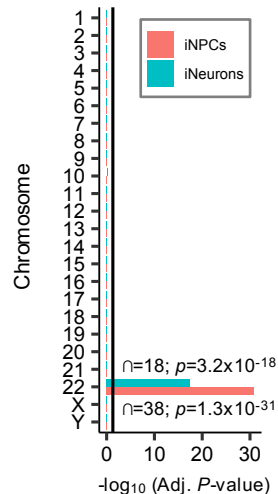

D

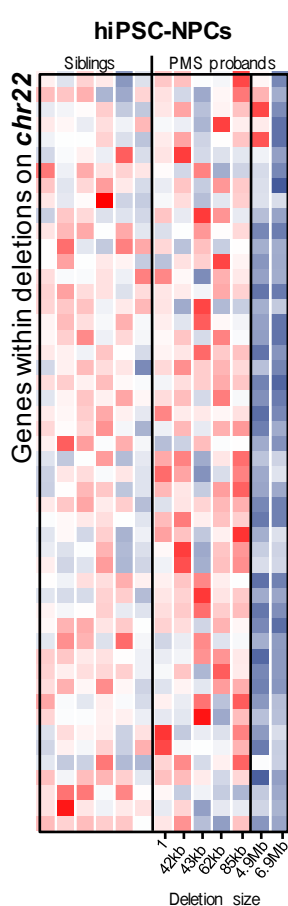

E

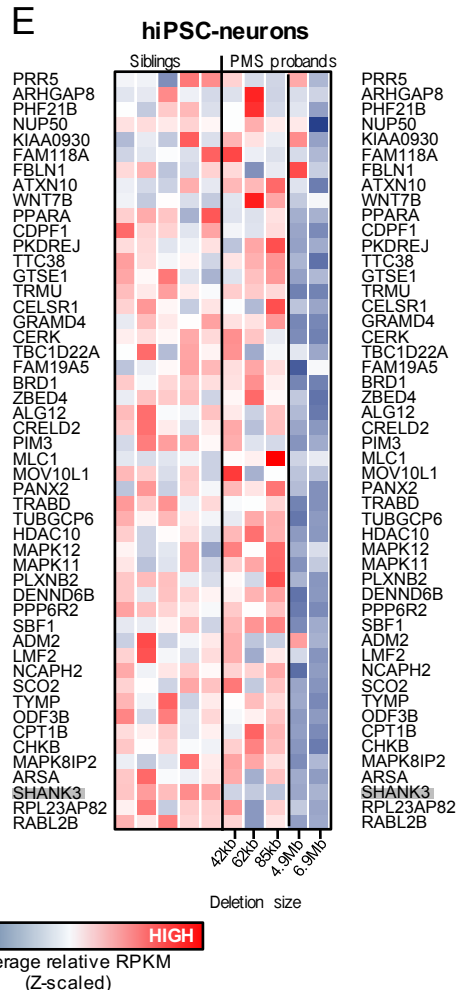

Supplement: Supplementary file 6 — Additional file 6: Figure S6. Variance explained by SHANK3 deletion size. (A) All genes affected by chr22 deletion in PMS proband from family 6 (4.9Mb deletion) are similarly affected in PMS proband from family 7 (6.9Mb deletion). (B) The linear mixed model framework of the varianceParition R package was used to compute the percentage of gene expression variance explained by SHANK3 deletion size in hiPSC-NPCs and hiPSC-neurons. (C) Genes with variance explained >50% by deletion size were examined for chromosomal enrichment, and strong enrichment for chromosome 22 was observed. The vertical black line indicates -log10 P-value < 0.05. Fifty unique genes were identified that varied by deletion size and mapped to chromosome 22, which were plotted on a heatmap using average expression values across all technical replicates for (D) hiPSC-NPCs and (E) hiPSC-neuronal samples. SHANK3 deletion sizes are displayed on the x-axis, and correspond to those present in Table 1. [file 13229_2020_355_MOESM6_ESM.pdf]

A

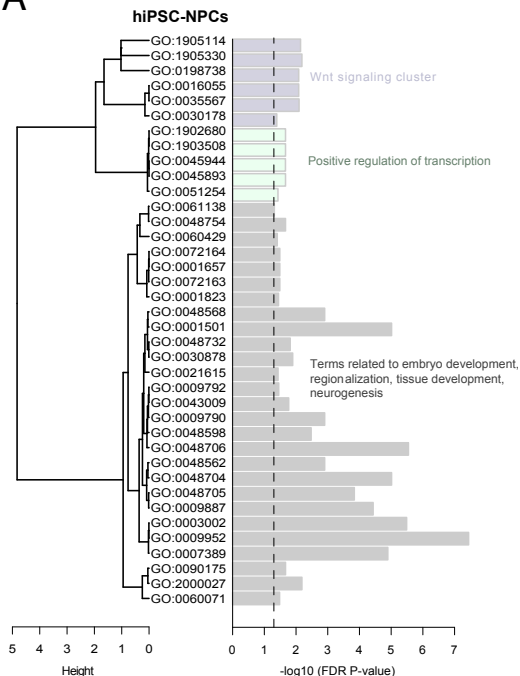

B

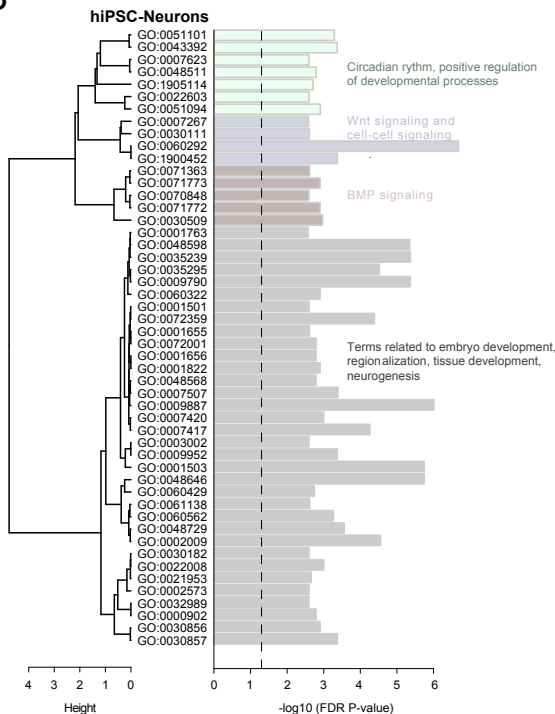

C

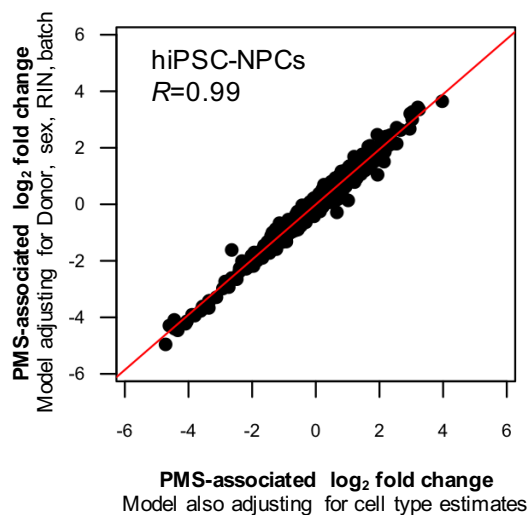

D

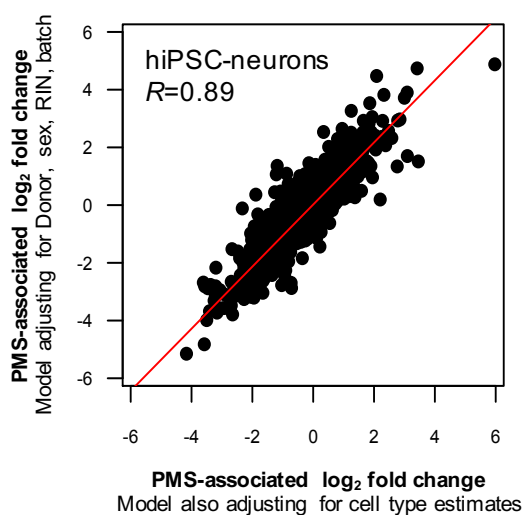

Supplement: Supplementary file 7 — Additional file 7: Figure S7. GO semantic similarity and incorporating cell type frequencies for differential expression. GO semantic similarity analysis was applied to examine shared/unique gene content among significantly under-expressed GO terms in (A) hiPSC-NPCs and (B) hiPSC-neurons. GO terms were then clustered based on ward and Euclidean distance and Ward’s clustering. The concordance of genome-wide PMS-associated log2 fold-changes were evaluated comparing two models: i) one model adjusting for sequencing batch, biological sex, RIN and individual donor as a repeated measure on the y-axis; and ii) a second model adjusting for the same factors plus predicted excitatory neuron cell type composition on the x-axis. Concordance was examined for both (C) hiPSC-NPCs and (D) hiPSC-neurons. [file 13229_2020_355_MOESM7_ESM.pdf]

A

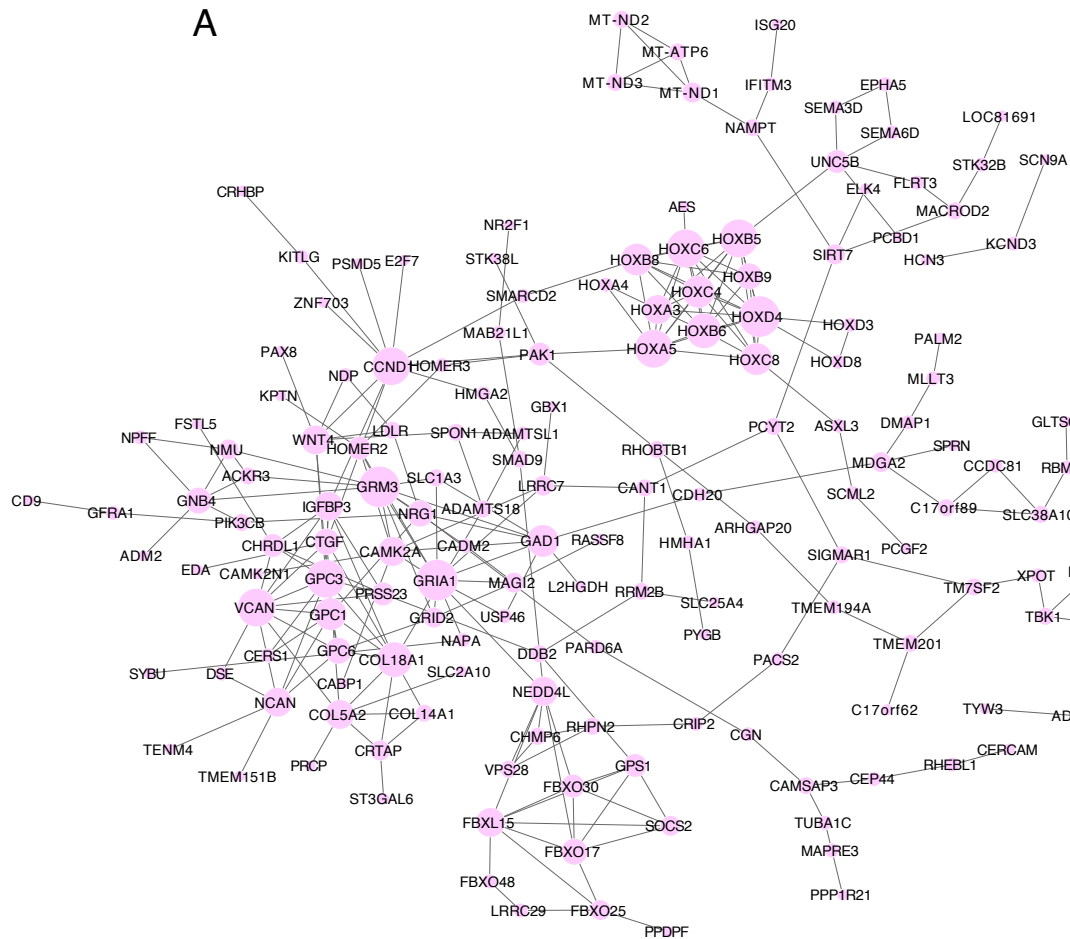

B

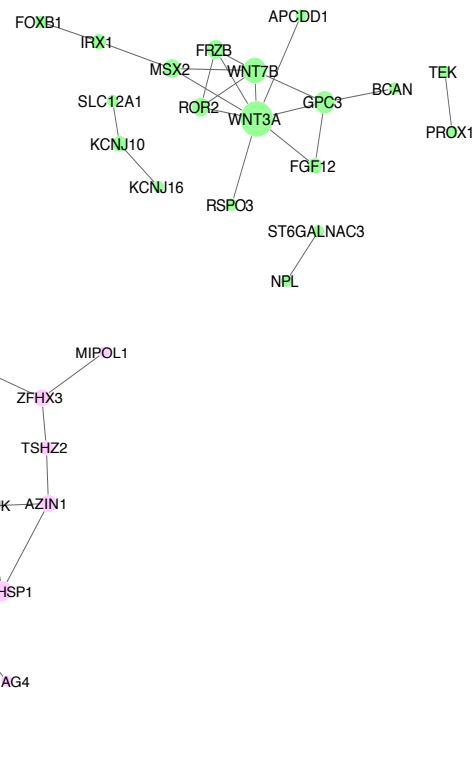

Supplement: Supplementary file 8 — Additional file 8: Figure S8. Protein-protein interaction network. Direct protein–protein interaction network of differentially expressed genes identified in (A) hiPSC-NPCs and (B) hiPSC-neurons. Nodes are scaled by their degree of overall connectivity in the network. [file 13229_2020_355_MOESM8_ESM.pdf]

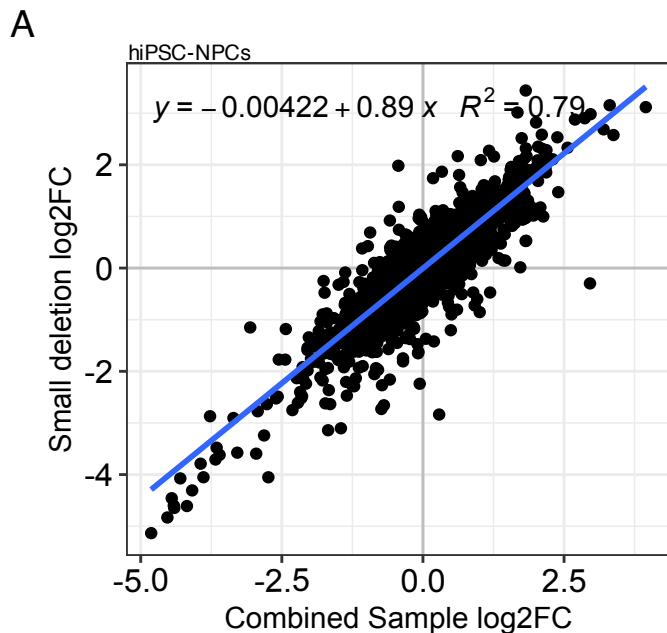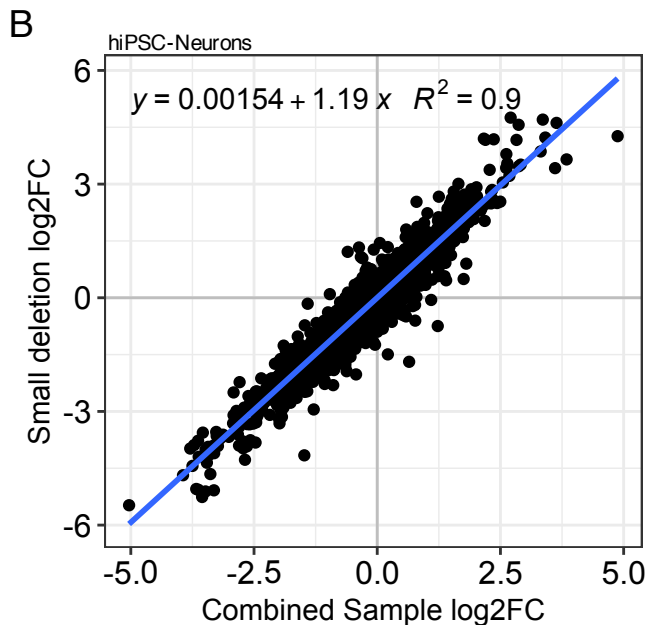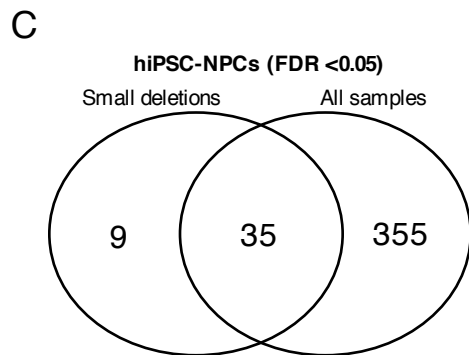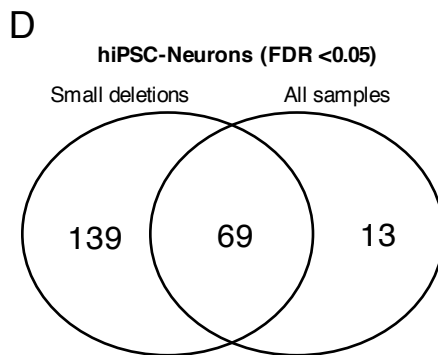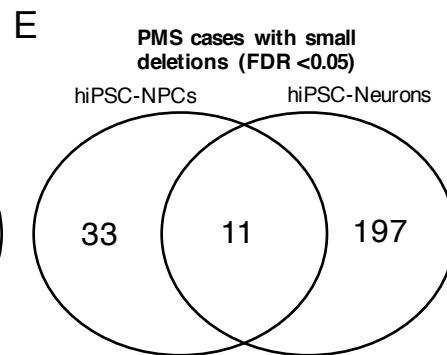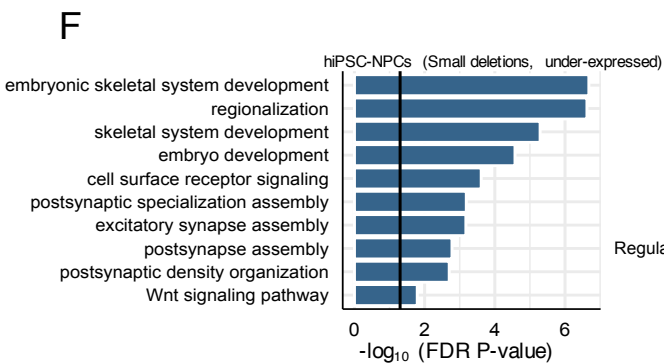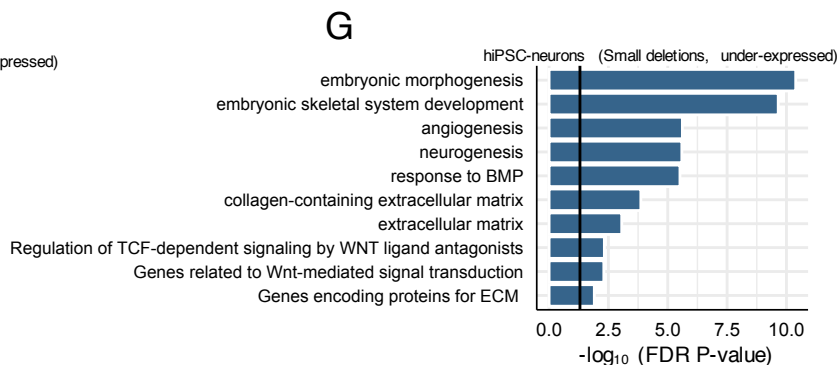

Supplement: Supplementary file 9 — Additional file 9: Figure S9. Differential expression in small deletion PMS cases. Genome-wide concordance of log2 fold-changes were examined for small deletion cases using (A) hiPSC-NPCs (4 PMS cases and 4 unaffected siblings, y-axis) and (B) hiPSC-neurons (3 PMS cases and 3 unaffected siblings, y-axis) relative to a pooled sample analysis as described in Fig. 2 (x-axes, respectively). Overlap of differentially expressed genes detected (C) in hiPSC-NPC small deletion cases compared to the pooled analysis (D) in hiPSC-neuron small deletion cases compared to the pooled analysis, and (E) between small deletion cases in hiPSC-NPCs and hiPSC-neurons. Gene ontology analysis of under-expressed genes in the PMS cases with small deletions were reported for (F) hiPSC-NPCs and (G) hiPSC-neurons. [file 13229_2020_355_MOESM9_ESM.pdf]

A

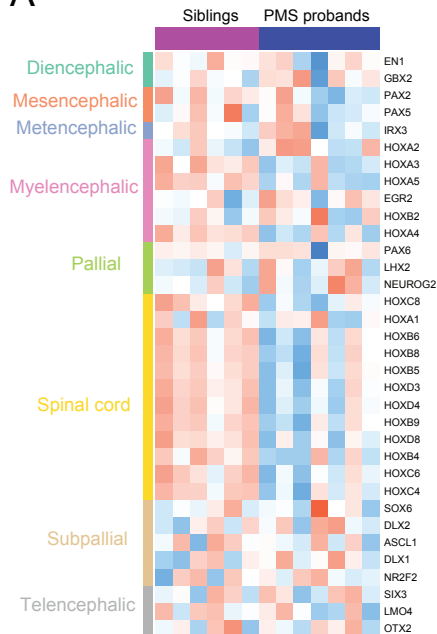

B

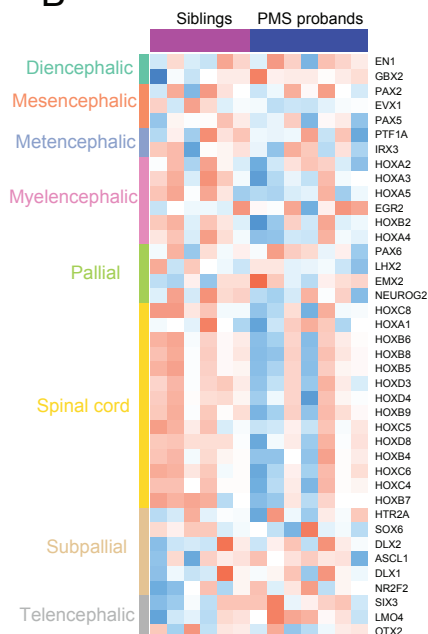

C

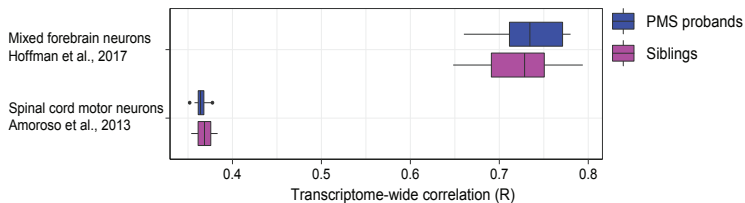

Supplement: Supplementary file 10 — Additional file 10: Figure S10. Regional marker genes. Marker genes covering eight different regional identities were evaluated for (A) hiPSC-NPCs and (B) hiPSC-neurons. Averaged expression values across 1-3 clones per donor for each marker were used to generate a heatmap. Regional identities (left of heatmap) and gene symbols (right of heatmap) are displayed. Heatmap colors are scaled from low (blue) medium (white) and high (red) expression. (C) Transcriptome-wide Pearson’s correlation between our hiPSC-neurons relative to two independent RNA-seq studies on forebrain hiPSC-neurons and spinal cord motor neurons, which are more posterior-like. [file 13229_2020_355_MOESM10_ESM.pdf]

A

## Scale independence

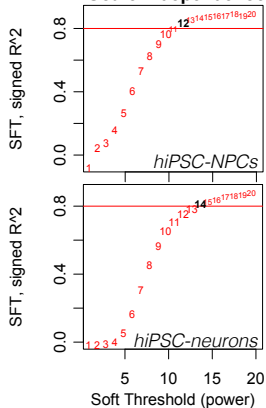

B

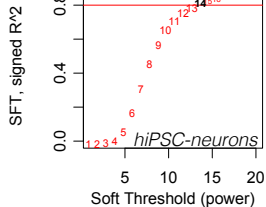

C

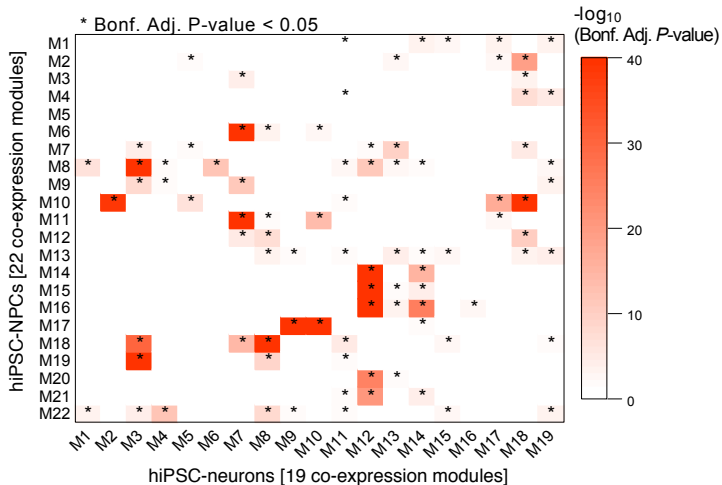

Supplement: Supplementary file 11 — Additional file 11: Figure S11. WGCNA module construction and overlap. The β-power defined for both (A) hiPSC-NPCsand (B) hiPSC-neurons in order to achieve scale free network topology for gene co-expression network construction. As a rule of thumb, β-power’s > 0.8 achieve scale free network topology, and a final β-power of 12 was used hiPSC-NPCs and a β-power of 14 for hiPSC-neurons. (C) Overlap analysis of co-expression modules defined based on hiPSC-NPCs and hiPSC-neurons. Significance of the overlap was tested using a one-sided Fisher’s exact test and corrected for multiple comparisons using Bonferroni procedure. Significant overlaps (*) are reported for overlaps displaying adjusted P<0.05. [file 13229_2020_355_MOESM11_ESM.pdf]

**A****hiPSC-neurons (6 weeks)**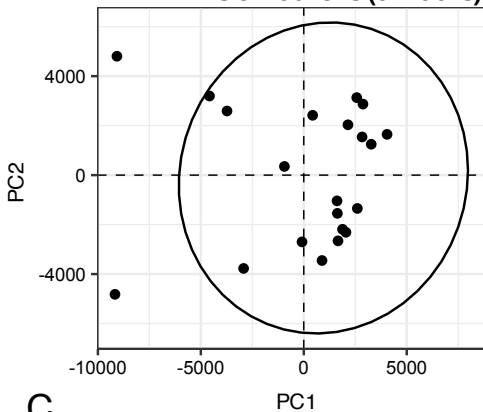**B****hiPSC-neurons (6 weeks)**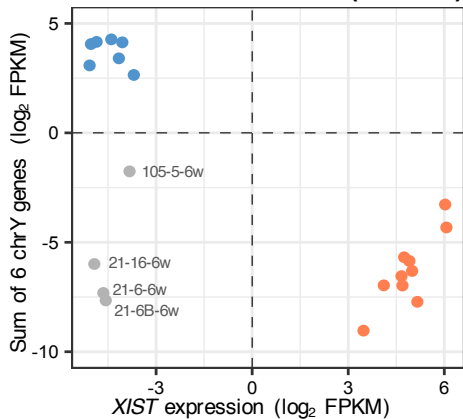**C**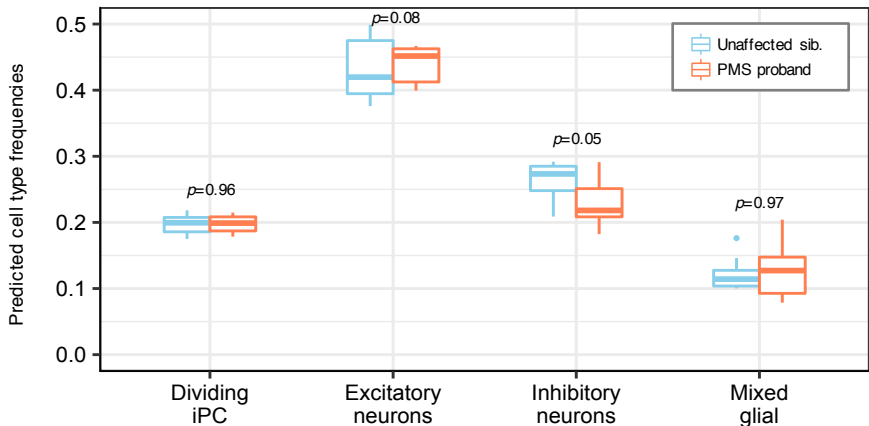

Supplement: Supplementary file 13 — Additional file 13: Figure S13. Data pre-processing using replication hiPSC-neurons. Principal component analyses were performed on RPKM values for (A) all replication set hiPSC-neurons at 6 weeks. Outliers beyond the 95% confidence intervals (black ellipse) were excluded from downstream analyses. (B) We also sought to identify samples that may have under-gone issues with X-inactivation and/or sample mislabeling by confirming that the reported biological sex is concordant with gene expression on chrX and chrY, which confirmed aberrant X-inactivation observed in hiPSC-NPCs sharing the same clone and induction (Supplemental Table 1). Samples with intermediate expression profiles were excluded from further analysis. (C) Cibersort cell type deconvolution analysis of global gene expression profiles estimated cell frequencies (y-axis) for four major cell types (x-axis) using a reference panel of single-cell RNA-sequencing data from the human fetal cortex. The predicted cellular proportions were compared between PMS probands and unaffected siblings using a Wilcox rank-sum test. [file 13229_2020_355_MOESM13_ESM.pdf]
