# Supplementary material for: Transcriptional signatures of participant-derived neural progenitor cells and neurons implicate altered Wnt signaling in Phelan-McDermid syndrome and autism
Source: Mol Autism. 2020 Jun 19;11:53. doi: 10.1186/s13229-020-00355-0 (PMC7304190; doi:10.1186/s13229-020-00355-0)
Supplement: Supplementary file 12 — Additional file 12: Figure S12. Overlap with other ASD hiPSC transcriptome studies. Convergence of differentially expressed genes (FDR <5%) in the current study with other ASD hiPSC transcriptome studies was assessed using a Fisher’s Exact Test (FET) and an estimated odds-ratio was computed in comparison to a genome-wide background set to 20,000. Significant overlaps are demarked with a red asterisks (*). All overlapping genes found in common with the current study were pooled and subjected to functional enrichment using FET and adjusting for genome background of 17,353 genes. Overlap of differentially expressed genes and functional annotation was performed using data from (A-B) Huang et al., 2019, (C-D) Wang et al., 2015, and (E-G) Chen et al., 2014, (H) Gigek et al., 2015, (I-J) Zheng et al., 2013, and (K-M) Deneault et al., 2018. Note that no functional enrichment was observed based on the overlap with (G) Chen et al., 2014 and (H) Gigek et al., 2015. To simply multiple overlaps, (K-L) the -log10 P-value (x-axis) is used to display the extent of significance based on gene expression perturbations associated with CRISPR/Cas9 knockout of 10 different ASD genes. [file 13229_2020_355_MOESM12_ESM.pdf]

A

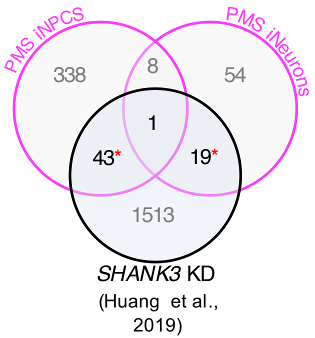


B

Enrichment based on overlapping features with *SHANK3* KD

| Gene set name | P-value | Adj. P-value |
|---|---|---|
| epithelial_cell_differentiation | 9.56E-07 | 0.0100 |
| cell_migration | 2.98E-06 | 0.0116 |
| canonical_Wnt_signaling_pathway | 3.32E-06 | 0.0116 |
| canonical_Wnt_signaling_pathway | 6.78E-06 | 0.0178 |
| endoplasmic_reticulum_lumen | 6.73E-05 | 0.0191 |
| smooth_endoplasmic_reticulum_lumen | 6.73E-05 | 0.0191 |
| cortical_endoplasmic_reticulum_lumen | 7.31E-05 | 0.0191 |
| perinuclear_endoplasmic_reticulum_lumen | 7.77E-05 | 0.0191 |
| rough_endoplasmic_reticulum_lumen | 9.48E-05 | 0.0191 |
| sarcoplasmic_reticulum_lumen | 0.000104437 | 0.0191 |

C

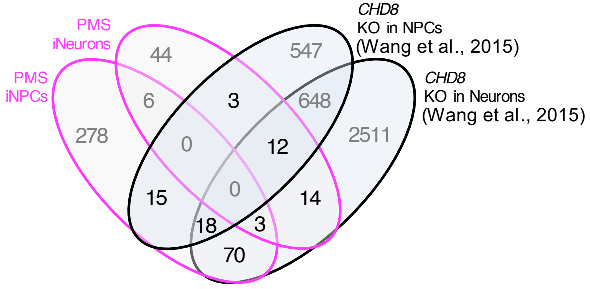


D

Enrichment based on overlapping features with *CHD8* KO

| Gene set name | P-value | Adj. P-value |
|---|---|---|
| galactosaminoglycan/glycosaminoglycan/mucopolysaccharide metabolism | 1.05E-08 | 2.77E-05 |
| Golgi_lumen | 1.52E-06 | 0.0016 |
| trans-Golgi_network_transport_vesicle_lumen | 2.92E-06 | 0.0016 |
| multicellular_organism_development | 2.04E-06 | 0.0019 |
| lysosomal_matrix | 8.16E-06 | 0.0022 |
| cortical_endoplasmic_reticulum_lumen | 2.44E-05 | 0.0032 |
| perinuclear_endoplasmic_reticulum_lumen | 2.66E-05 | 0.0032 |
| endolysosome_lumen | 2.25E-05 | 0.0032 |
| proteinaceous_extracellular_matrix | 0.000205759 | 0.0188 |
| SMAD_protein_complex | 0.000885591 | 0.0495 |
| canonical_Wnt_signaling_pathway | 0.000205759 | 0.0544 |

E

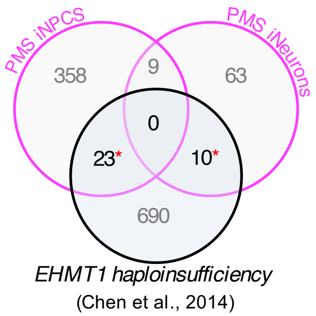


F

Enrichment based on overlapping features with *EHMT1* haploinsufficiency

| Gene set name | P-value | Adj. P-value |
|---|---|---|
| Golgi_lumen | 9.77E-06 | 0.00770 |
| trans-Golgi_network_transport_vesicle_lumen | 1.43E-05 | 0.0077 |
| hyaluronic_acid_binding | 3.10E-06 | 0.0106 |
| galactosaminoglycan/glycosaminoglycan/mucopolysaccharide metabolism | 7.58E-06 | 0.0197 |
| biofilm_matrix_organization | 4.32E-05 | 0.0292 |
| cellulose_microfibril_organization | 4.32E-05 | 0.0292 |
| extracellular_matrix_organization | 4.32E-05 | 0.0292 |
| basement_membrane_organization | 4.44E-05 | 0.0292 |
| fibronectin_fibril_organization | 4.44E-05 | 0.0292 |
| extracellular_matrix_assembly | 5.07E-05 | 0.0296 |
| collagen_fibril_organization | 5.77E-05 | 0.0319 |
| central_nervous_system_neuron_differentiation | 6.53E-05 | 0.0343 |
| chondroitin_sulfate_biosynthetic_process | 7.31E-05 | 0.0366 |

G

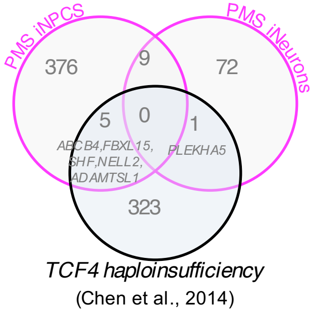


H

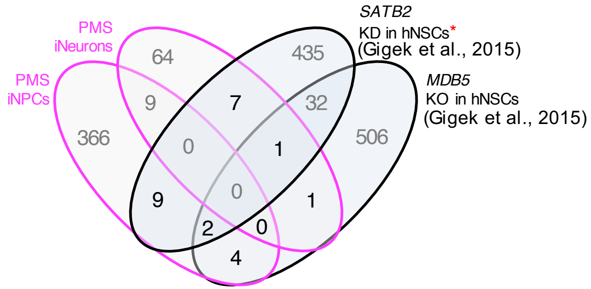


I

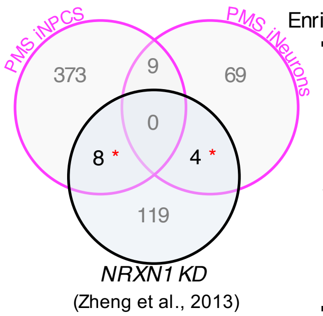


J

Enrichment based on overlapping features with NRXN1 KD

| Gene set term | P-value | Adj. P-value |
|---|---|---|
| canonical_Wnt_signaling_pathway | 0.0003 | 0.4296 |
| beta-catenin_destruction_complex_disassembly | 0.0005 | 0.4296 |
| receptor_internalization_involved_in_Wnt signaling | 0.0011 | 0.756 |
| Golgi_lumen | 0.0014 | 0.7816 |
| trans-Golgi_network_transport_vesicle_lumen | 0.0017 | 0.7816 |
| L-glutamate_import | 0.0034 | 1 |
| central_nervous_system_neuron_differentiation | 0.0037 | 1 |
| L-glutamate_import | 0.0046 | 1 |
| ion_channel_complex | 0.0052 | 1 |

K

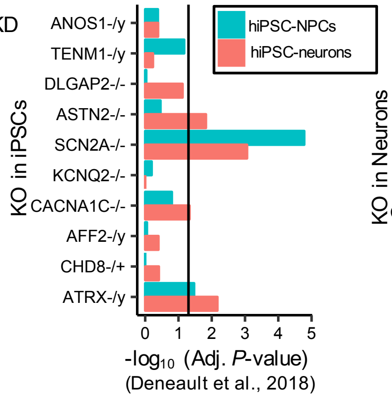


L

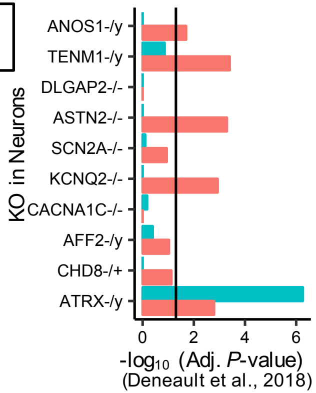


M

Enrichment based on overlapping features with ATRX-/y

| Gene set name | P-value | Adj. P-value |
|---|---|---|
| cell_migration_involved_in_sprouting_angiogenesis | 0.0001 | 0.6967 |
| cerebellar_granule_cell_differentiation | 0.0002 | 0.4866 |
| positive_regulation_of_Wnt_signaling_pathway | 0.0002 | 0.8067 |
| glutamate_receptor_activity | 0.0003 | 0.4866 |
| contractile_vacuole_discharge | 0.0004 | 0.8067 |
| regulated_exocytosis | 0.0004 | 0.4866 |
| ionotropic_glutamate_receptor_activity | 0.0005 | 0.4866 |
| endocytic_vesicle_membrane | 0.0006 | 0.0904 |
| synapse_assembly | 0.0009 | 0.6358 |
| calcium_ion_regulated_exocytosis | 0.0011 | 0.8067 |
| NMDA_glutamate_receptor_activity | 0.0012 | 0.8067 |
